# Harnessing tumor immune checkpoints for autoimmune disease therapeutics

**DOI:** 10.3389/fimmu.2026.1725372

**Published:** 2026-09-10

**Authors:** Hanmei Yang, Ping Zhang, Yi Wu, Na Xie, Guobo Shen

**Affiliations:** 1 Department of Biotherapy, Cancer Center and State Key Laboratory of Biotherapy, West China Hospital and West China School of Basic Medical Sciences and Forensic Medicine, Sichuan University, Chengdu, China; 2 Department of Oncology, Chengdu Integrated TCM & Western Medicine Hospital, Chengdu, China

**Keywords:** autoimmune diseases, immune checkpoints, immune tolerance, immunoregulation, inhibitory receptors, targeted therapy

## Abstract

Immune checkpoint (IC) pathways, originally identified in T-cell exhaustion in cancer, are increasingly recognized as key modulators of immune tolerance in autoimmune diseases (ADs). In ADs, these molecules function more accurately as inhibitory receptors (IRs) that fine-tune, rather than inducing classical exhaustion phenotypes, autoreactive immune cells. Unlike oncology, where checkpoint blockade enhances immunity, AD therapy aims to restore tolerance through IC agonism or immune reset approaches (e.g., CAR-T cells). This review synthesizes recent advances in targeting CTLA-4, PD-1, LAG-3, TIM-3, and TIGIT. Clinical success with CTLA-4-Ig (abatacept) and emerging efficacy of PD-1 agonists (e.g., peresolimab) provide proof-of-concept that augmenting inhibitory signaling can ameliorate autoimmunity. However, outcomes remain context-dependent, reflecting interplay between effector and regulatory subsets and tissue-specific environments. Mechanistic challenges, including multivalent receptor engagement, ligand complexity, and signaling heterogeneity (e.g., ITIM/ITSM phosphorylation), limit current approaches. Insights from immune-related adverse events (irAEs) highlight shared pathways of dysregulation and inform risk-benefit considerations. A major translational gap persists between acute transplant models and chronic autoimmunity, and validated predictive biomarkers are lacking. Beyond T cells, B cells, dendritic cells, and innate populations expand therapeutic opportunities. Future progress will require multi-omics profiling, biomarker-driven stratification, and next-generation agonist design. IC-targeted strategies hold promises but demand context-aware, mechanistically informed development for broad clinical impact in ADs.

## 1 Introduction

**Figure 1:**
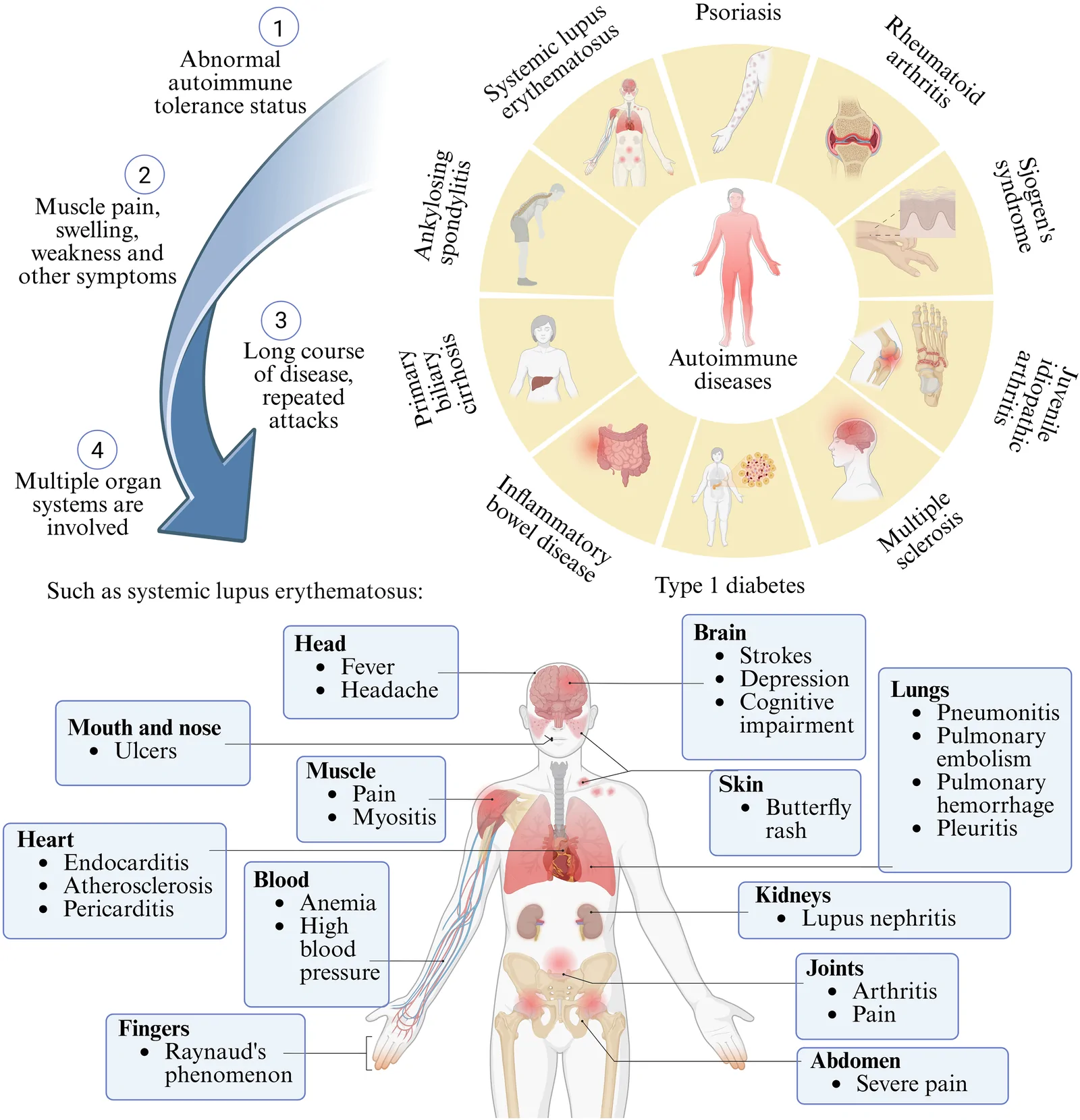
Types and characteristics of ADs. There are many types of ADs, the main common diseases are systemic lupus erythematosus (SLE), psoriasis, rheumatoid arthritis (RA), Sjogren’s syndrome (SjS), juvenile idiopathic arthritis (JIA), multiple sclerosis (MS), type 1 diabetes (T1D), ulcerative colitis (UC), Crohn’s disease (CD), primary biliary cirrhosis, and ankylosing spondylitis. Symptoms of ADs vary by disease and affected organ, and some ADs are often chronic and recurring and affect certain types of tissues throughout the body, such as blood vessels, cartilage, or skin. The resulting inflammation and tissue damage can cause pain, joint deformities, weakness, and itching.

Autoimmune diseases (ADs) are a heterogeneous group of disorders characterized by aberrant immune activation, whereby immune cells erroneously attack the body’s own healthy tissues and organs (1), as illustrated in **Figure 1**. To date, more than one hundred distinct ADs have been identified, collectively affecting approximately 10% of the global population (2, 3). Further insights from the Global Burden of Disease Study show that the prevalence of immune-mediated inflammatory diseases, including psoriasis, multiple sclerosis (MS), inflammatory bowel disease (IBD), and rheumatoid arthritis (RA), is highest among older adults (0.73% in those aged ≥55 years) and children/adolescents (0.96% in those aged <20 years), with substantial variations across sex and sociodemographic index (4). A pronounced gender disparity exists, with women disproportionately affected. Indeed, the prevalence of ADs in women exceeds the combined incidence of heart disease and breast cancer, underscoring their status as a major public health burden (5). Under physiological conditions, the immune system functions to detect and eliminate pathogens such as bacteria and viruses, thereby maintaining homeostasis. In ADs, however, self-tissues are misidentified as foreign, leading to chronic inflammation and tissue damage (6, 7).

Immune checkpoints (ICs) are specialized regulatory mechanisms that modulate immune signaling, thereby preventing excessive immune activation, unchecked tumor growth, disruption of self-tolerance, and the onset of autoimmunity (8). However, the term “immune checkpoint” has distinct connotations depending on the disease context, a nuance that is critical for understanding therapeutic logic and has been a recurring theme in recent literature (9). In oncology, ICs refer to inhibitory pathways (e.g., programmed cell death protein 1/ligand (PD-1/PD-L1), T-lymphocyte–associated antigen 4/ligands (CTLA-4/CD80/CD86) that tumors exploit to evade immune destruction; consequently, cancer immunotherapy aims to block these checkpoints to unleash anti-tumor immunity (10, 11). In ADs, by contrast, the same molecules function as “inhibitory receptors” (IRs) that fine-tune, rather than fully exhaust, autoreactive immune cells. As recently articulated, immune checkpoints act as “dimmer switches” that calibrate activation, differentiation, and contraction in response to antigen strength, co-signals, cytokines, and tissue context. Dysregulation in autoimmunity typically manifests as four overlapping patterns: insufficient inhibitory tone, elevated co-stimulation, metabolic or epigenetic pathway remodeling, and mismatched tissue versus blood profiles (9, 12–14). This conceptual distinction has direct therapeutic implications: whereas oncology uses checkpoint blockade, AD therapy requires the opposite approach—agonizing co-inhibitory receptors or blocking co-stimulatory pathways to restore immune tolerance.

These checkpoints are essential in the context of ADs, as they inhibit self-reactive immune responses and fine-tune the activity of immune effector cells (15, 16). Conversely, dysregulation of IC pathways contributes to the initiation and progression of ADs (17). Elucidating the role of ICs is thus critical for advancing our understanding of immune homeostasis and for developing effective diagnostic and therapeutic approaches for ADs.

Targeting ICs represents a paradigm shift in precision medicine for ADs. While IC-based therapies have been widely employed in oncology, where blockade of co-inhibitory checkpoints or activation of co-stimulatory checkpoints enhances antitumor immunity (18), the therapeutic logic in ADs is reversed. Because ADs result from aberrant immune hyperactivity against self-tissues, therapeutic strategies aim to restore tolerance by activating co-inhibitory pathways or inhibiting co-stimulatory ones. By suppressing antigen-presenting cell (APC) and T cell interactions, immune cell activity can be attenuated, thereby reducing inflammation and tissue injury, alleviating clinical symptoms, and achieving therapeutic benefit (19). Conventional treatments for ADs, including cyclophosphamide, interleukin inhibitors, and long-term corticosteroid use, are often limited by adverse effects such as heightened infection risk, immunosuppression, and osteoporosis (20). In contrast, checkpoint-targeted therapies hold promise for alleviating AD symptoms while maintaining sufficient immune function to prevent infections and disease recurrence, thus achieving a more balanced immune state.

Before delving into the specific roles of individual checkpoint molecules, it is important to place checkpoint-mediated regulation within the broader framework of immune tolerance. The immune system employs multiple hierarchical layers to prevent autoimmunity, ranging from central tolerance mechanisms that eliminate highly self-reactive lymphocytes during development to peripheral tolerance mechanisms that control those that escape into the periphery (21, 22). Central tolerance occurs in the thymus (for T cells) and bone marrow (for B cells), where lymphocytes bearing strongly self-reactive antigen receptors are deleted through clonal deletion during V(D)J recombination and somatic hypermutation (23, 24). Peripheral tolerance includes mechanisms such as anergy, ignorance, and suppression by regulatory T cells (Tregs), as well as checkpoint-mediated regulation via CTLA-4, PD-1, LAG-3 (lymphocyte activation gene 3), TIM-3 (T-cell immunoglobulin and mucin domain–3), and TIGIT (25, 26). It is important to recognize that checkpoint dysregulation is not necessarily the primary causative defect in most polygenic, multifactorial ADs; rather, it likely represents a modulatory layer superimposed on more fundamental defects in central or peripheral tolerance. This recognition does not diminish the therapeutic potential of targeting IRs but places realistic expectations on patient selection and combination strategies. Notably, rare monogenic defects in checkpoint molecules, such as CTLA-4 haploinsufficiency, can directly cause severe, early-onset autoimmunity in humans, providing compelling genetic validation of the checkpoint agonism strategy (27, 28).

The modulation of ICs provides a promising avenue for the precision treatment of ADs and warrants further in-depth investigation. This review aims to delineate the functional roles of ICs, their involvement in the pathogenesis of ADs, and emerging strategies for their therapeutic targeting. We also explore the potential of combining IC-based interventions with other therapeutic modalities and highlight key unresolved issues including the translational gap between transplant models and chronic autoimmunity, the lack of predictive biomarkers, and the need for long-term safety data on tumor surveillance. Collectively, this work seeks to provide a comprehensive reference for advancing IC–based therapies in ADs and to offer novel insights for clinical translation.

## 2 ICs in immune regulation

### 2.1 Conceptual evolution and characteristics of ICs

**Figure 2:**
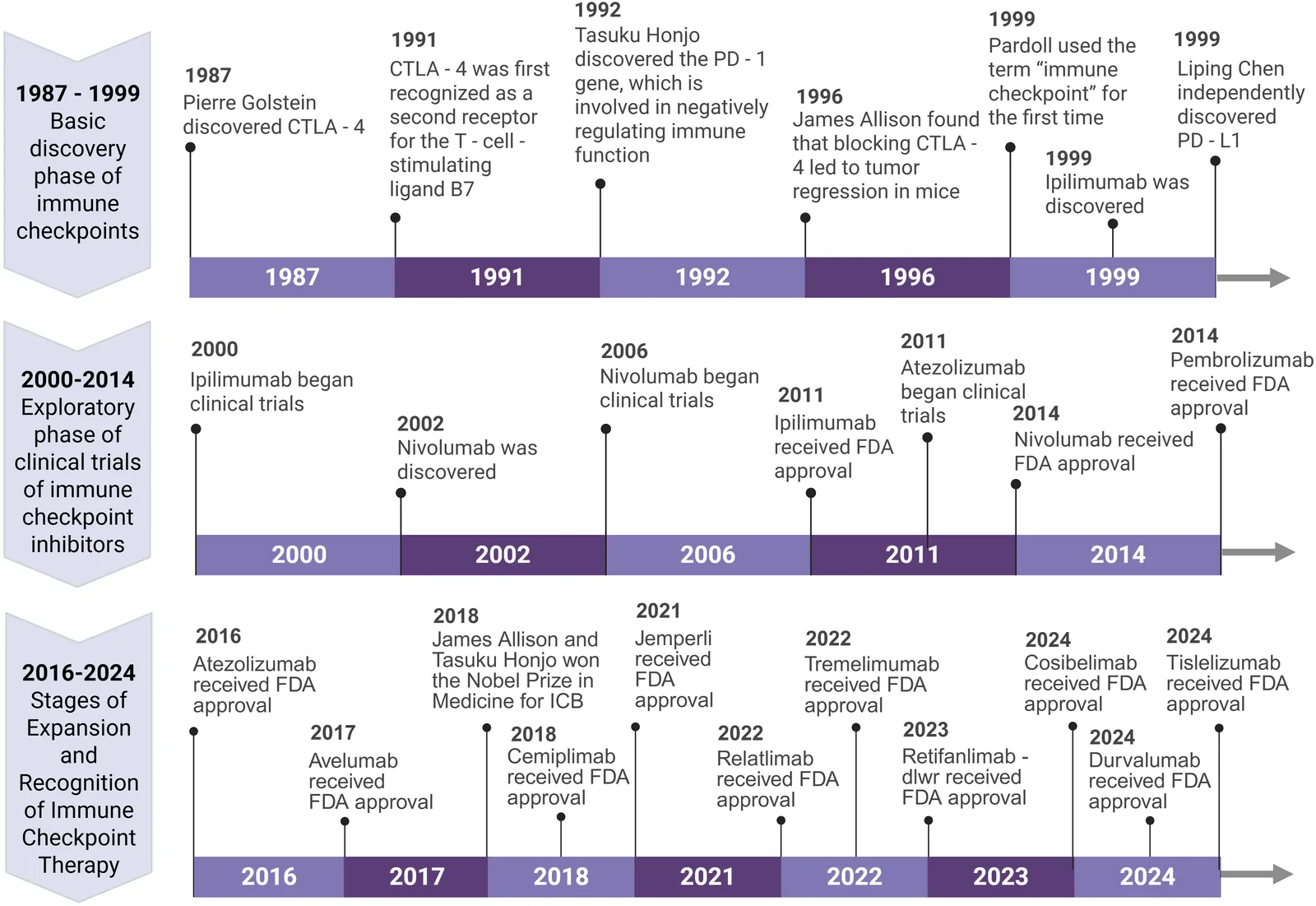
Key time points and milestones in the process of ICs from discovery to clinical application are as follows. In the first phase, from 1987-1999, key molecules related to ICs, such as CTLA-4, PD-1, PD-L1, etc., were discovered and initially recognized, and the term “immune checkpoints” was proposed for the first time. In the second phase, from 2000 to 2014, various IC inhibitors were discovered and began to enter clinical trials, and some of the drugs began to obtain FDA approval, marking an important transition from laboratory to clinical application of IC therapy. In the third phase, from 2016 to 2024, more and more IC inhibitors were approved by the FDA, and the scope of application continued to expand, while the related research results were highly recognized, such as the awarding of the Nobel Prize.

In the fields of oncology and immunology, the concept of ICs has evolved from fundamental research discoveries into a cornerstone of modern therapeutic strategies. To illustrate this trajectory, **Figure 2** presents a timeline of key milestones, from the identification of relevant molecules to the clinical approval of IC-based therapies. The conceptual framework of ICs began to take shape in the early 1990s, encompassing mechanisms of both tolerance and self-tolerance (29). The notion of “tolerance” was first articulated by Macfarlane Burnet in 1948, who proposed that immune inertia toward self-antigens is acquired during development (30). By 1999, Pardoll formally introduced the term “immune checkpoint” in the context of exploiting autoimmunity for cancer treatment (31). Since then, the concept has been substantially refined and expanded by subsequent immunologists, including Korman, Peggs, and Allison, thereby accelerating both the study of ICs and the clinical development of immunotherapies (32).

In the context of autoimmunity, the molecules commonly referred to as “ICs” in oncology function more accurately as inhibitory receptors (IRs) that fine-tune autoreactive immune cells rather than inducing classical exhaustion (25, 33). This distinction has direct therapeutic implications and is elaborated in the Introduction. These IRs are expressed across diverse immune cell subsets, including activated T cells, natural killer (NK) cells, dendritic cells (DCs), and others (33) (**Table 1**). Importantly, their expression patterns and levels vary depending on immune cell type, tissue microenvironment, and pathological state. For example, in certain tumor cells, overexpression of programmed death-ligand 1 (PD-L1) serves as a mechanism of immune evasion (34).

**Table 1:** Classic inhibitory immune checkpoint receptors in autoimmune diseases as discussed in this article: mechanisms, cellular context, and translational considerations.

IC/IR	Family	Major cell types	Ligands	Mechanism of action	Role in ADs	Therapeutic strategy	Key limitation	References
CTLA-4	B7–CD28	Activated T cells, Tregs	CD80/CD86	Competes with CD28; trans-endocytosis of CD80/CD86; limits costimulation	Maintains peripheral tolerance; deficiency → systemic autoimmunity	CTLA-4–Ig (abatacept, belatacept)	Context-dependent efficacy; abatacept ≠ full endogenous CTLA-4 function	(71, 214)
PD-1	B7–CD28	Effector T cells, Tregs, B cells, DCs	PD-L1, PD-L2	ITIM/ITSM phosphorylation → SHP-2 recruitment; inhibits TCR signaling	Regulates effector/regulatory balance; dysfunction linked to ADs	PD-1 agonists (e.g., peresolimab), PD-L1–Fc	Requires receptor clustering; dual effects on effector vs Tregs	(67, 215)
LAG-3	Ig superfamily	T cells, Tregs, NK cells	MHC-II, FGL1	Inhibits TCR signaling; modulates Treg function	Controls immune activation; synergizes with PD-1	LAG-3 agonists; combination with PD-1	Context-dependent Treg effects; limited biomarkers	(141)
TIM-3	TIM family	T cells, DCs, macrophages, NK cells	Galectin-9, HMGB1, CEACAM-1, PtdSer	Multi-ligand signaling; regulates T-cell functional attenuation and innate activation	Modulates both adaptive and innate responses	Experimental agonists	Ligand promiscuity; unclear dominant pathway in humans	(216)
TIGIT	Ig superfamily	T cells, Tregs, NK cells	CD155, CD112	Competes with CD226; inhibitory signaling in T/NK cells	Regulates Treg stability and Th17 balance	Emerging agonists	Functional redundancy with PD-1; variable expression patterns	(25, 217)
VISTA	B7 family	T cells, myeloid cells (DCs, monocytes, neutrophils)	PSGL-1, VSIG-3	Functions as both ligand and receptor; suppresses T-cell activation and cytokine production	Maintains peripheral tolerance; dysregulation promotes Th1/Th17 responses	Agonists (autoimmunity), antagonists (oncology)	Dual receptor/ligand role complicates targeting; context-dependent signaling	(218)
BTLA	Ig superfamily	T cells, B cells, DCs	HVEM	ITIM-mediated inhibitory signaling; dampens TCR activation	Maintains immune tolerance; implicated in rheumatic diseases	BTLA agonists (early clinical exploration)	Redundant pathways (e.g., PD-1); limited clinical efficacy so far	(166)
CD40	TNF–TNFR	B cells, DCs, macrophages, T cells	CD40L (CD154)	Activates NF-κB signaling; enhances APC activation and T–B interaction	Promotes autoantibody production; central in lupus and RA	CD40/CD40L blockade	Thromboembolic risk (anti-CD40L); pleiotropic immune activation	(219)
OX40	TNF–TNFR	Activated T cells, Tregs, APCs, NK cells	OX40L	Enhances T-cell survival, proliferation, cytokine production	Promotes inflammation; blockade reduces autoimmunity in models	OX40L blockade or modulation	Can disrupt Treg stability; strong pro-inflammatory potential	(220)
ICOS	B7–CD28	Tfh cells, activated T cells, B cells, DCs	ICOSL	Activates PI3K–AKT–mTOR pathway; supports Tfh differentiation	Drives germinal center responses and autoantibody production	ICOS/ICOSL blockade	Dual role in effector and regulatory responses	(221)

A major conceptual advance in recent years has been the recognition that T-cell activation and regulation cannot be adequately explained by the classic “two-signal” hypothesis (Signal 1: TCR-MHC; Signal 2: CD28 co-stimulation). Contemporary immunology has expanded this framework to a “four-signal” model that integrates additional layers of control (35, 36):

- Signal 1: Antigen recognition—TCR binding to peptide–MHC complex on APCs.
- Signal 2: Co-stimulation—CD28 binding to CD80/CD86 (positive) or CTLA-4 competing for the same ligands (negative).
- Signal 3: Cytokine signaling—Polarizing cytokines (e.g., IL-2, IL-12, IFN-γ, IL-4, IL-17, TGF-β) that drive T-cell differentiation into distinct effector or regulatory subsets.
- Signal 4: Metabolic and nutrient sensing—Metabolic cues such as glucose availability, amino acid levels, oxygen tension, and AMPK/mTOR pathway activity that profoundly influence T-cell fate and function.

Importantly, IC signaling does not act in isolation but integrates extensively with cytokine and metabolic pathways. For example, PD-1 engagement inhibits the PI3K/Akt/mTOR axis, thereby suppressing glycolysis and promoting fatty acid oxidation—a metabolic switch that favors Treg differentiation over effector T-cell proliferation (37, 38). Conversely, persistent TCR signaling in the absence of adequate co-stimulation can induce anergy, a state of functional unresponsiveness that is reinforced by IC signaling (39).

Another critical consideration often overlooked in reviews is the pharmacological challenge of agonizing inhibitory receptors. Unlike blocking a receptor, which typically requires only high-affinity binding to prevent ligand engagement, agonism of ICs to restrain excessive immune activation is exponentially more complex (40). Effective agonism often requires multivalent ligand crosslinking to induce clustering of the receptor, followed by phosphorylation of immunoreceptor tyrosine-based inhibition motifs (ITIMs) or tyrosine-based switch motifs (ITSMs) in the cytoplasmic tail. These phosphorylated motifs recruit phosphatases such as SHP-1 and SHP-2, which then dephosphorylate downstream signaling molecules (e.g., Zap70, PI3K) to attenuate T-cell activation (41). Most current IC agonists are bivalent monoclonal antibodies that may not achieve sufficient receptor clustering to trigger full signaling, potentially explaining the high attrition rate in clinical development (42). This pharmacological reality underscores the need for novel engineering approaches such as multivalent antibody formats, bispecific molecules, or nanoparticle-based platforms to achieve robust IR agonism in AD therapy.

### 2.2 Roles of ICs in maintaining immune homeostasis

ICs exert their regulatory functions through ligand–receptor interactions that activate or inhibit immune cell activity (43) (**Table 1**). For T-cell activation, co-stimulatory signals are indispensable. Full T-cell activation, expansion, and differentiation require multiple signals, as discussed in Section 2.1. Specifically, Signal 1 is delivered by the TCR–CD3 complex binding to the major histocompatibility complex II (MHC-II) on APCs (44), Signal 2 is provided by APC-expressed co-stimulatory molecules, such as CD28, 4-1BB, and CD40 (cluster of differentiation 40), engaging their corresponding receptors on T cells, and Signals 3 and 4 (cytokine and metabolic cues) further fine-tune the outcome (36, 45). Disruption of this signaling balance can provoke aberrant immune activation and autoimmune responses (46).

Co-inhibitory molecules, including CTLA-4, PD-1, LAG-3 and TIM-3, represent evolutionarily conserved regulatory mechanisms that safeguard against immune overactivation (47). Dysregulation of these inhibitory pathways contributes to the pathogenesis of ADs. For instance, CTLA-4 polymorphisms have been linked to heightened susceptibility to type 1 diabetes (T1D) (48), MS (49), and systemic lupus erythematosus (SLE) (50). Similarly, PD-1 polymorphisms are associated with increased risk of RA (51) and MS (52).

An important layer of complexity is that many ICs are co-expressed on both effector and regulatory immune subsets, and their net effect depends on the cellular context. For example, PD-1 is expressed not only on activated effector T cells but also on Tregs, where PD-1 signaling can enhance suppressive capacity (53). Consequently, the balance between PD-1^+^ effector T cells and PD-1^+^ regulatory T cells may influence the efficacy of a PD-1 agonist, and this balance has been suggested as a potential predictor of therapeutic response (54). Similarly, CTLA-4 is constitutively expressed on Tregs and contributes to their suppressive function, whereas its expression on effector T cells is induced upon activation (55, 56). These contextual differences underscore the need for cell-type–specific analyses when evaluating IC-targeted therapies.

Beyond T cells, ICs are also expressed on other immune cell types that play critical roles in AD pathogenesis. B cells contribute to autoimmunity through autoantibody production, antigen presentation, and cytokine secretion, but they express relatively low levels of canonical co-inhibitory ICs (e.g., PD-1, CTLA-4), which may limit their direct responsiveness to current IC agonists (57). In contrast, DCs and NK cells often show high expression of ICs such as TIGIT, LAG-3, and TIM-3, making them underexplored yet promising targets. Modulating ICs on DCs could promote tolerogenic DC phenotypes, while on NK cells it might regulate cytotoxicity and cytokine production—opening new therapeutic avenues beyond T-cell–centric approaches (58–60).

Thus, ICs function as a double-edged sword, simultaneously governing immune activation and inhibition, with profound implications for both autoimmunity and cancer. For example, IC inhibitors can disrupt tumor–immune cell communication, thereby restoring T-cell activation and proliferation. Anti–CTLA-4 antibodies (e.g., ipilimumab) exemplify this mechanism by blocking inhibitory signaling and enhancing T-cell activity (61). Conversely, CTLA-4 fusion proteins (e.g., CTLA4-Ig) mimic CTLA-4 function by competitively binding to B7 family molecules (CD80 and CD86), thereby modulating T-cell activation (62). However, these approaches carry risks, including immune-related adverse events (irAEs) (see Section 5). In the context of ADs, maintaining the delicate equilibrium of IC signaling is therefore crucial, as its dysregulation contributes both to autoimmunity (63) and, conversely, to tumorigenesis (64). Ultimately, the therapeutic modulation of ICs is highly context-dependent and must be tailored to the specific clinical setting.

## 3 Therapeutic targeting of ICs in ADs

Targeted ICs therapy has emerged as a promising avenue in the management of ADs. Originally developed in oncology to overcome tumor-induced immunosuppression and restore anti-tumor immunity, IC modulation is now being investigated as a strategy to rebalance dysregulated immune responses in autoimmunity. However, as highlighted in Section 2.1, the pharmacological challenge of agonizing ICs is substantially greater than that of blocking them. Most current IC agonists are bivalent monoclonal antibodies that may not achieve sufficient receptor clustering to trigger full signaling, a factor that has contributed to high attrition rates in clinical development (42). This section critically evaluates the evidence for targeting individual ICs in ADs, emphasizing mechanistic distinctions, clinical successes and failures, and unresolved challenges.

ADs are frequently driven by excessive co-stimulatory signaling or insufficient co-inhibitory signaling. Consequently, therapeutic interventions aim either to attenuate co-stimulation or to reinforce co-inhibition (65). Two principal approaches are under investigation: (i) depleting IC-positive cells using drugs, antibodies, or immunotoxins, and (ii) enhancing inhibitory signaling through fusion proteins, monoclonal antibodies, or nucleic acid-based agents (66).

**Figure 3:**
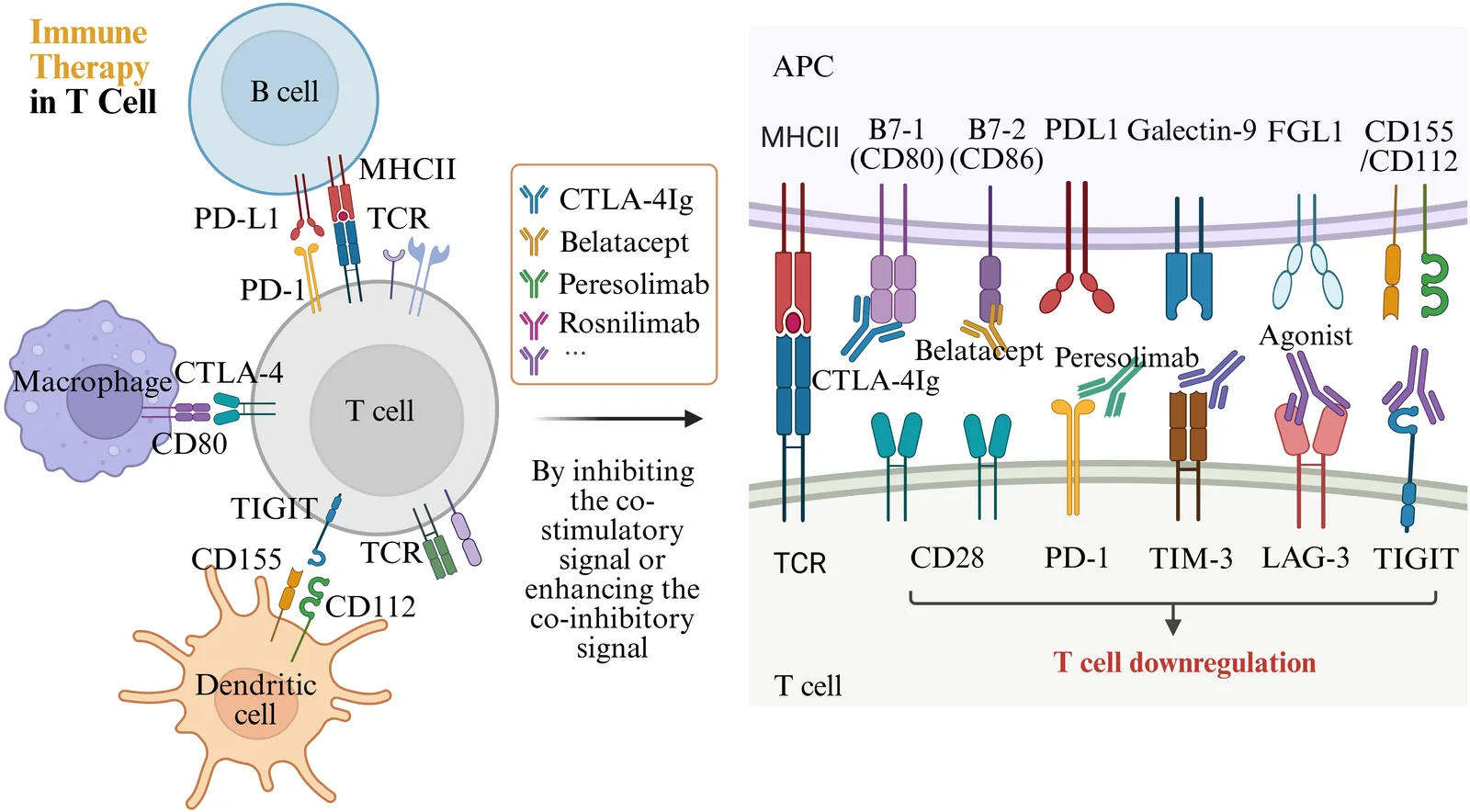
Approaches for T-cell immunotherapy. Antigen-presenting cells (APC), major histocompatibility complex (MHC), and T cells are three important components of the immune system. APC are immune cells that can process and present antigens to T cells. These include macrophages, dendritic cells, and B cells. MHC exists on the surface of APC, and when an antigen binds to MHC, an antigen MHC complex is formed, which can be recognized by specific T cells, and the MHC complex binds to the T cell antigen receptor (TCR) to activate the immune response. Nevertheless, this step is insufficient for the full activation of T cells, which requires the involvement of co-stimulatory signals. Drugs can target ICs to block T cell activation pathways or enhance inhibitory pathways, limiting T cell immune responses, avoiding excessive inflammation and damage, and inhibiting ADs.

A key insight from oncology is that blockade of co-inhibitory checkpoints can enhance immune responses but is also associated with irAEs, many of which resemble autoimmune manifestations; this relationship is discussed in detail in Section 5 (67). In contrast, AD therapy leverages the opposite principle, agonism or reinforcement of co-inhibitory pathways, to restore immune tolerance and limit tissue damage. Co-inhibitory checkpoints such as CTLA-4, PD-1, TIM-3, LAG-3 and TIGIT therefore represent attractive therapeutic targets, with several already undergoing clinical evaluation (**Figure 3**). In the following sections, we discuss the roles of these checkpoints in AD pathogenesis and highlight their translational potential as therapeutic targets, with a focus on comparative analysis, mechanistic limitations, and future directions.

### 3.1 Cytotoxic T lymphocyte antigen-4

CTLA-4 (also known as CD152) was the first IC identified. Discovered by Pierre Golstein and colleagues in 1987, it was later shown by Leach, Krummel, and Allison in 1996 that CTLA-4 blockade in mice could induce transplant rejection, establishing its role as a negative regulator of T-cell responses (61, 68). CTLA-4 shares ligands (CD80 and CD86) with CD28 but binds with higher affinity, thereby competing with CD28 and inhibiting T-cell activation and proliferation.

Genetic studies underscore *CTLA-4*’s relevance to autoimmune disease susceptibility. GWAS and meta-analyses have consistently linked the rs231775 (+49 A>G) polymorphism to increased RA risk, with the G allele associated with diminished *CTLA-4* function and elevated T-cell activation (69). Additionally, the rs3087243 variant in the 3’-UTR has been correlated with higher T1D risk in multiple populations, supported by meta-analytic data (70). These findings reinforce the genetic basis for targeting CTLA-4 signaling pathways as a therapeutic strategy in ADs.

A critical mechanistic distinction must be made between the endogenous CTLA-4 receptor and the therapeutic agent abatacept (CTLA-4-Ig). Endogenous CTLA-4 is a transmembrane protein expressed on T cells that actively regulates immune responses through trans-endocytosis—the physical removal of CD80 and CD86 ligands from the surface of APCs, thereby preventing their engagement with CD28 (71). Abatacept, in contrast, is a soluble chimeric protein composed of the CTLA-4 extracellular segment attached to the Fc portion of human IgG1. It acts as a competitive steric sink, binding to CD80/CD86 with higher affinity than CD28 but lacking the ability to actively remove ligands or transduce intracellular signals (55, 72). This mechanistic difference has important implications: abatacept provides passive blockade of co-stimulation, whereas endogenous CTLA-4 actively shapes the APC surface and contributes to Treg function.

Compelling human genetic evidence supports the CTLA-4 agonism strategy. Heterozygous germline loss-of-function mutations in *CTLA4*, leading to haploinsufficiency, cause a primary immune dysregulation disorder characterized by multi-organ autoimmunity, hypogammaglobulinemia, lymphoproliferation, and increased susceptibility to infections (27). Clinical manifestations include autoimmune cytopenia, enteropathy, interstitial lung disease, and neurological involvement, with a highly variable phenotypic spectrum (27, 73). Importantly, treatment with abatacept (CTLA-4-Ig), which functionally compensates for the defective endogenous CTLA-4, has demonstrated remarkable clinical efficacy in patients with CTLA-4 haploinsufficiency, inducing sustained remission of autoimmune manifestations, resolution of hypogammaglobulinemia, and improvement in neuroinflammatory lesions (74, 75). A landmark case report documented a 15-year-old girl with severe multifocal neuroinflammation due to a *de novo CTLA4* variant who achieved brilliant clinical and radiological responses after abatacept dose adjustment (75). These findings directly validate that pharmacological enhancement of CTLA-4 function can reverse established autoimmunity in humans, bridging the conceptual gap between genetic checkpoint deficiency and therapeutic checkpoint agonism.

**Figure 4:**
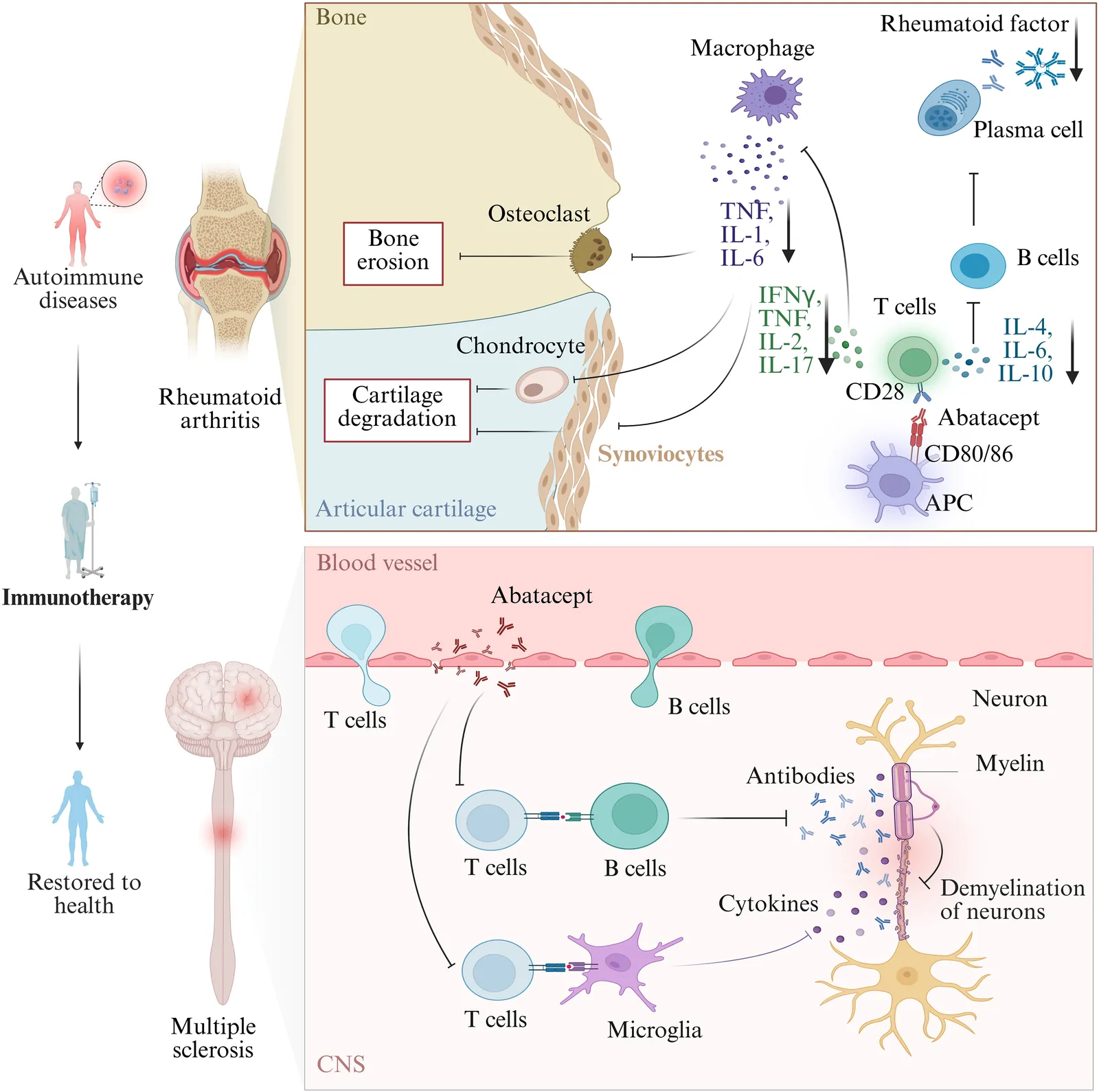
Mechanisms of IC drugs in the treatment of ADs such as RA and MS. The pathogenesis of RA is multifactorial, but T cells play a pivotal role in its development and progression. Activated effector T cells, particularly Th1 and Th17 subsets, stimulate macrophages to produce pro-inflammatory mediators such as TNF-α, IL-1, and IL-6, thereby driving synovial inflammation, bone erosion, and cartilage degradation. IC drugs such as Abatacept improve RA by binding to CTLA-4, inhibiting T-cell activity, and reducing the release of pro-inflammatory factors from macrophages. MS is a complex disease that affects the central nervous system (CNS). The immune system initiates an attack on myelin, a protective coating and insulating material surrounding nerve fibers in the brain and spinal cord. This results in impaired nerve signaling. IC drugs cross the blood-brain barrier and enter the CNS, reducing the interaction between T cells and B cells and reducing the release of antibodies. On the other hand, it reduces the interaction of T cells with microglia and reduces the release of cytokines. Together, they inhibit neuronal demyelination.

Abatacept (CTLA4-Ig) is the best-characterized therapeutic agent targeting CTLA-4 and has been widely applied in AD therapy (69, 76). It mimics checkpoint signaling while retaining immunoglobulin properties. By competitively binding CD80/CD86 on APCs with greater affinity than CD28, it suppresses T-cell co-stimulation, reduces effector cytokine secretion (IL-2, IFN-γ), limits effector T-cell proliferation, and modulates Treg activity (**Figure 4**). As Tregs are critical for immune tolerance, abatacept’s ability to enhance Treg function offers therapeutic potential in mitigating aberrant immune responses. Clinically, abatacept is approved for RA, psoriatic arthritis, and juvenile idiopathic arthritis (JIA) (77, 78). It is used as a second-line therapy in refractory RA and as a first-line option for rapidly progressive disease. Multiple clinical trials (NCT00095173, NCT01835470, NCT01844518) have confirmed its efficacy and safety in JIA, with subcutaneous formulations demonstrating particular benefit in adolescents (79). In Japanese patients with JIA, intravenous abatacept improved remission rates with favorable tolerability and minimal severe adverse events (80). Long-term studies confirmed durable efficacy and safety, with patients tolerating therapy for up to seven years (81). Beyond arthritis, abatacept has been investigated in T1D, a T-cell–mediated disease characterized by progressive β-cell destruction. Early intervention with abatacept preserved residual insulin secretion, delayed C-peptide decline, and improved HbA1c outcomes in new-onset T1D, with benefits persisting up to three years after diagnosis (82–84). Belatacept, a CTLA4-Ig variant with approximately tenfold greater activity, has been applied in transplantation to reduce rejection risk and is being explored in T1D as a potential alternative to conventional immunosuppressants with high toxicity (85).

However, CTLA-4-targeted therapy has shown only modest effectiveness in other autoimmune settings, underscoring the heterogeneity of these diseases. In ulcerative colitis (UC) and Crohn ‘s disease (CD), abatacept showed no benefit and, in severe UC, worsened disease outcomes (86). Similarly, results in SLE have been inconsistent; while early studies suggested benefit, later trials failed to demonstrate efficacy, and in rare cases abatacept has been associated with SLE onset in RA patients (87, 88). Investigations in MS, primary biliary cirrhosis, lupus nephritis, SjS, and ankylosing spondylitis remain experimental, with outcomes pending further validation (**Table 2**). These failures suggest that CTLA-4 agonism may be most effective in diseases where T-cell co-stimulation via CD28/CD80/CD86 is a dominant pathogenic driver, whereas in diseases with prominent B-cell, innate immune, or alternative T-cell pathways, additional or different targeting strategies may be required.

**Table 2:** Clinical trials of drugs targeting CTLA-4, PD-1, TIM-3, LAG-3 in various ads.

Immune checkpoint	Medicine	Disease	NCT number	Research purpose	Phase	Sponsor	Results
CTLA-4	CTLA-4-Ig(abatacept)	MS	NCT01116427	Efficacy	Phase 2	National Institute of Allergy and Infectious Diseases (NIAID)	In patients with MS, abatacept has been well tolerated with the majority of adverse events being mild
			NCT00035529	Preliminary efficacy	Phase 2	Bristol-Myers Squibb	Terminated
		SLE arthritis	NCT02429934	Safety and effectiveness	Phase 1, Phase 2	University of California, Los Angeles	Terminated (Response rate in placebo group for primary outcome was 100% on interim analysis)
		Lupus nephritis	NCT00430677	Efficacy and safety	Phase 2, Phase 3	Bristol-Myers Squibb	Terminated (Terminated due to failure to meet the primary efficacy endpoint in the Short-term Period)
		SjS	NCT02067910	Efficacy and safety	Phase 3	University Medical Center Groningen	Completed
		SLE	NCT02270957	Efficacy	Phase 2	Oklahoma Medical Research Foundation	Completed
		Ankylosing spondylitis	NCT00558506	Efficacy and safety	Phase 2	Charite University, Berlin, Germany	Unknown
		Ulcerative colitis	NCT00410410	Efficacy and safety	Phase 3	Bristol-Myers Squibb	Abatacept is safe but not effective in the treatment of moderate to severe CD or UC
		Primary biliary cirrhosis	NCT02078882	Efficacy and safety	Phase 4	Christopher Bowlus, MD	Completed
		Psoriatic arthritis	NCT04610476	To assess the effect of dose reduction	Phase 3	University of Erlangen-Nürnberg Medical School	Phase3 study is recruiting
		Psoriasis Vulgaris	NCT01999868	Efficacy of abatacept after ustekinumab treatment	Phase 2	National Institute of Allergy and Infectious Diseases (NIAID)	It is not effective in preventing the recurrence of psoriasis after the withdrawal of Ustekinumab
			NCT00306878	Safety, pharmacokinetics, immunogenicity	Phase 1	Bristol-Myers Squibb	Completed
			NCT00277225	Single-dose pharmacokinetics, immunogicity and safety	Phase 1	Bristol-Myers Squibb	Completed
			NCT00287547	Safety, pharmacokinetics, clinical activity and immunogenicity	Phase 2	Bristol-Myers Squibb	Completed
		RA	NCT02504268	Efficacy and safety	Phase 3	Bristol-Myers Squibb	Although the primary endpoint of the study was not met, abatacept was beneficial for early RA
			NCT00484289	Safety	Phase 3	Bristol-Myers Squibb	In Japanese patients with RA, intravenous abatacept has been shown to be safe and consistently effective for more than 3 years
			NCT01001832	Efficacy, pharmacokinetics, safety, and immunogenicity	Phase 2, Phase 3	Bristol-Myers Squibb	In Japanese patients with RA, abatacept is well tolerated and effective, and has an acceptable safety profile and low immunogenicity
			NCT03882008	Efficacy	Phase 4	University of Washington	Unknown
			NCT01339481	To characterize CD86 receptor occupancy	/	Astellas Pharma Inc	Completed
		JIA	NCT01835470	Efficacy, safety, pharmacokinetics and immunogenicity	Phase 3	Bristol-Myers Squibb	Intravenous abatacept is effective and well tolerated.
			NCT03841357	Efficacy	Phase 3	Duke University	Phase3 study is recruiting
			NCT01844518	Pharmacokinetics, efficacy and safety	Phase 3	Bristol-Myers Squibb	The minimum target therapeutic concentration of 10 μg/ml does not affect patient safety
			NCT00095173	Efficacy and safety	Phase 3	Bristol-Myers Squibb	Abatacept is effective, safe and a reasonable treatment
			NCT03769558	To determine the incidences of infections and malignancies among JIA patients treated with abatacept	/	Bristol-Myers Squibb	Completed
	CTLA4-IgG4m(RG2007)	Multiple sclerosis	NCT00076934	Safety	Phase 1	National Institute of Allergy and Infectious Diseases (NIAID)	Completed
		SLE arthritis	NCT00094380	Efficacy and safety	Phase 1, Phase 2	National Institute of Allergy and Infectious Diseases (NIAID)	Completed
	CTLA-4-IgG1(belatacept)	T1D	NCT00276250	Efficacy and safety	Phase 2	Emory University	Completed
			NCT00501709	Efficacy and safety	Phase 1, Phase 2	University of California, San Francisco	Completed
			NCT00468403	Efficacy and safety	Phase 2	National Institute of Allergy and Infectious Diseases (NIAID)	Completed
		RA	NCT00279760	Safety, initial clinical activity, immunogenicity	Phase 1, Phase 2	Bristol-Myers Squibb	Completed
		Vitiligo	NCT02281058	Efficacy	Phase 1	Brigham and Women’s Hospital	Unknown
PD-1	Rosnilimab (ANB030)	RA	NCT06041269	Safety, tolerability, and efficacy	Phase 2	AnaptysBio, Inc.	Phase2 study is recruiting
		Ulcerative colitis	NCT06127043	Safety, tolerability, and efficacy	Phase 2	AnaptysBio, Inc.	Phase2 study is recruiting
		Alopecia areata	NCT05205070	Efficacy and safety	Phase 2	AnaptysBio, Inc.	Unknown
	Peresolimab	RA	NCT05516758	Safety and efficacy	Phase 2	Eli Lilly and Company	Active, not recruiting
	JNJ-67484703	RA	NCT04985812	Safety and tolerability	Phase 1	Janssen Research & Development, LLC	Completed
	CC-90006	Plaque-type psoriasis	NCT03337022	Safety, tolerability, Pharmacokinetics, Pharmacodynamics, and immunogenicity	Phase 1	Celgene	Completed
LAG-3	GSK2831781	Plaque psoriasis	NCT02195349	Safety, tolerability, Pharmacokinetics and Pharmacodynamics	Phase 1	GlaxoSmithKline	GSK2831781 is pharmacologically active and provides encouraging early evidence of clinical efficacy in psoriasis
		Ulcerative colitis	NCT03893565	Safety, tolerability, efficacy and dose-response	Phase 2	GlaxoSmithKline	GSK2831781 Failed to Reduce Inflammation in the Colonic Mucosa. The study ended early

In summary, CTLA-4–targeted therapy represents a major advance in autoimmune immunotherapy. Abatacept has shown robust efficacy in RA, JIA, and psoriatic arthritis, and promising results in T1D, but limited effectiveness in other autoimmune conditions highlights the heterogeneity of immune pathophysiology. A central unresolved issue is how to extend the efficacy of CTLA-4-based therapies beyond currently responsive indications. Can higher dosing or more potent CTLA-4 agonists (e.g., belatacept) broaden the therapeutic scope? Which biomarkers might predict a favorable response? Could combination with other IC agonists or cytokine inhibitors overcome resistance? Answering these questions will require disease-specific clinical studies, long-term safety assessments, and a shift toward personalized strategies.

### 3.2 Programmed cell death receptor-1

PD-1 (CD279) was first identified in 1992 by Tasuku Honjo in T-cell hybridomas undergoing apoptosis, a feature that inspired its name (89). Belonging to the CD28 family of immunoglobulin superfamily receptors and structurally related to CTLA-4, PD-1 was later shown by Honjo and Freeman (2000–2001) to interact with its ligands PD-L1 (CD274, B7-H1) and PD-L2 (CD273, B7-DC), thereby establishing it as a pivotal inhibitory checkpoint (90, 91). PD-1 engagement limits T-cell expansion, cytokine release, and cytotoxicity, thus sustaining peripheral tolerance and preventing immune-mediated tissue damage (92).

Genetic and experimental evidence highlights the importance of this pathway in autoimmunity. In mice, *PD-1* deficiency is associated with lupus-like glomerulonephritis, dilated cardiomyopathy, and heightened susceptibility to experimental autoimmune encephalomyelitis (EAE) (93–96). In humans, variants within the PD-1/PD-L1 axis, such as rs4143815 in the *PD-L1* promoter, are enriched in SLE and may reduce checkpoint activity (97). Altered PD-1 expression patterns have also been linked to disease: T cells from psoriatic arthritis patients display elevated PD-1, while in RA, PD-1 expression on peripheral T cells inversely correlates with disease activity, suggesting a compensatory regulatory function (98, 99). Consistent with this regulatory role, systemic activation of the PD-1/PD-L1 axis via PD-L1-Ig has been shown to restrain Th17 cell formation in multiple organs, including the spleen and kidney, thereby attenuating lupus-like nephritis in SLE-prone mice (100). Collectively, these findings establish PD-1 as a key inhibitory checkpoint whose dysfunction contributes to AD initiation and progression.

Several conceptual refinements are essential for understanding PD-1 targeting in ADs. First, PD-1 is co-expressed on both effector T cells and Tregs) On Tregs, PD-1 signaling can enhance suppressive capacity (53). Thus, the overall outcome of PD-1 agonist therapy may be dictated by the balance between PD-1^+^ effector T cells and PD-1^+^ Tregs, a ratio that has been suggested as a candidate predictive marker (101). Second, PD-1 engagement inhibits the PI3K/Akt/mTOR axis, thereby suppressing glycolysis and promoting fatty acid oxidation—a metabolic switch that favors Treg differentiation over effector T-cell proliferation (37, 102). Third, the concept of “tissue tolerance” is critical: local PD-L1 upregulation in tissues (e.g., placenta, eye, transplanted organs) restricts effector T-cell entry. Systemically administered PD-1 agonists differ fundamentally from tissue-specific endogenous PD-L1 upregulation, and this distinction has implications for both efficacy and safety (91).

Therapeutic strategies targeting PD-1 have been under exploration for two decades. Early efforts in the early 2000s focused on PD-1 agonists for RA, though no agent advanced beyond preclinical stages. More recently, peresolimab (LY3462817), a selective PD-1 agonist, demonstrated clinical efficacy. By activating PD-1 signaling, peresolimab attenuated lymphocyte activation and expansion, reduced disease activity in phase 2 RA trials, and exhibited a safety profile comparable to placebo (103). However, as noted in Section 2.1, most current PD-1 agonists are bivalent antibodies that may not achieve sufficient receptor clustering to trigger full ITIM/ITSM phosphorylation and SHP-1/2 recruitment, which could explain the high attrition rate in clinical development (42). Other agonists under clinical evaluation include rosnilimab (phase 2, alopecia areata), JNJ-67484703 (phase 1, RA), and CC-90006 (phase 1, psoriasis), though development has been inconsistent, with some programs stalled. Additional molecules such as RTX-002, PT627, and MB151 remain preclinical. While PD-1 agonism represents a promising approach for ADs, concerns remain regarding potential impairment of tumor surveillance, underscoring the need for long-term safety data (40).

Alternative strategies exploit PD-1 ligands. Increasing platelet PD-L1 expression reverses autoimmune diabetes in preclinical models by suppressing autoreactive immunity and enhancing Treg activity (104). PD-L1-Fc fusion proteins, which mimic endogenous PD-L1, have demonstrated efficacy across animal models of colitis, SLE, and psoriasis by modulating DCs, reducing autoantibody production, and improving tissue pathology (105–108). However, clinical validation in humans is lacking.

A distinct approach involves depletion of PD-1–positive pathogenic cells. Novel immunotoxins, combining anti-PD-1 antibody fragments with bacterial toxin domains, selectively eliminated PD-1^+^ populations in murine models of autoimmune diabetes and experimental autoimmune encephalomyelitis, delaying disease onset, alleviating pathology, and preserving adaptive immunity (109, 110). Notably, these constructs retained antitumor immune surveillance in preclinical testing, suggesting favorable therapeutic selectivity.

Critical limitations of PD-1–targeted therapies in ADs must be acknowledged. First, although early preclinical and clinical studies suggest activity in RA and psoriasis, robust efficacy across other ADs, including SLE, MS, and T1D, remains to be established. Second, the limited advancement of multiple PD-1 agonist programs (e.g., CC-90006 in psoriasis) highlights the pharmacological challenge of achieving durable receptor agonism in humans, reflecting the intrinsic difficulty of recapitulating physiological inhibitory signaling with exogenous agonists. Third, the potential long-term risk of impaired tumor surveillance remains a theoretical concern, as most clinical trials to date have had short follow-up durations. Fourth, the lack of validated predictive biomarkers, such as PD-1^+^ effector/Treg ratio, baseline PD-1 expression, or functional restraint signatures, continues to hinder patient stratification and response prediction. Collectively, these challenges underscore the gap between biological rationale and clinical translation in PD-1 agonist–based immunotherapy.

In summary, PD-1 represents a central inhibitory checkpoint in immune regulation and a promising therapeutic target in autoimmunity. While peresolimab and other agonists provide the first clinical evidence of efficacy, complementary ligand-based and cell-depleting strategies are emerging. A key challenge is that effective PD-1 agonism requires precise receptor clustering and context-dependent signaling, which remains difficult to achieve pharmacologically. Key priorities include engineering multivalent or bispecific PD-1 agonists for robust receptor clustering, validating predictive biomarkers (e.g., PD-1^+^ effector/Treg ratio) to guide patient selection, and testing combinations with other IC agonists. Ongoing studies will be critical to determine the long-term safety, durability, and disease-specific applicability of PD-1–targeted interventions.

### 3.3 T cell immunoglobulin and mucin domain-containing protein 3

The therapeutic efficacy of CTLA-4 and PD-1 blockade has been widely demonstrated (111, 112). However, treatment with monoclonal antibodies targeting these checkpoints is often accompanied by drug resistance and adverse reactions (113, 114). These limitations have prompted the search for additional IC targets. Among them, TIM-3 has emerged as a promising candidate due to its involvement in chronic viral infections, tumor immune tolerance, and T cell dysfunction (115, 116). Growing evidence suggests that co-blockade of TIM-3 and PD-1 not only helps overcome resistance to PD-1 inhibitors but also provides synergistic therapeutic benefits (117, 118).

Within autoimmune settings, TIM-3 serves as a key regulator of both innate and adaptive immunity. TIM-3 signaling can influence antigen presentation, as demonstrated by its ability to downregulate MHC-II expression, thereby ameliorating autoimmune encephalomyelitis (119). Conversely, *TIM-3* deficiency increases susceptibility to EAEs, underscoring its importance in maintaining immune homeostasis (120). Evidence also links TIM-3 to SLE, although its precise pathogenic role remains incompletely defined (121). Notably, its ligand Galectin-9 has been shown to attenuate lupus-like manifestations in mice by inducing apoptosis of Th1 and Th17 effector cells, thereby reducing their abundance (122). Additional mechanisms include Galectin-9–mediated suppression of anti-dsDNA antibody production and amelioration of renal pathology through CD44-dependent inhibition of plasmacytoid DCs, a process independent of TIM-3 signaling (123).

TIM-3 has also been implicated in the regulation of Th17-driven inflammation in psoriasis and psoriatic arthritis. Th17 cells, which produce proinflammatory cytokines such as IL-17, express TIM-3, and engagement of this receptor appears to inhibit their activity, thereby dampening inflammatory responses (124). Furthermore, TIM-3 activation may promote T cell apoptosis, further contributing to the attenuation of disease progression (125). These findings highlight the therapeutic potential of TIM-3 modulation, although the underlying mechanisms remain to be fully elucidated.

A major challenge in targeting TIM-3 therapeutically arises from its ligand promiscuity. TIM-3 binds at least four distinct ligands, Galectin-9, HMGB1, phosphatidylserine (PtdSer), and CEACAM-1, each triggering distinct signaling outcomes (126). HMGB1, a damage-associated molecular pattern (DAMP) molecule, is typically released during tissue injury and inflammation; its interaction with TIM-3 on DCs has been shown to suppress nucleic acid-mediated innate immune responses, but the same alarmin also exerts pro-inflammatory effects through other receptors such as TLR4 and RAGE (127). CEACAM-1, another TIM-3 ligand, forms a heterodimer with TIM-3 on activated T cells and is required for TIM-3-mediated inhibition; however, CEACAM-1 also participates in homophilic adhesion and can modulate immune responses in a context-dependent manner (128). The functional consequences of TIM-3 ligation thus vary depending on which ligand is engaged, the cell type, and the inflammatory milieu. This ligand complexity raises important safety concerns for TIM-3-targeted therapies: agonists designed to engage Galectin-9 may inadvertently block HMGB1 signaling or trigger pro-inflammatory pathways through CEACAM-1 interactions, potentially exacerbating rather than alleviating autoimmune inflammation. Furthermore, the net effect of TIM-3 modulation may differ between cell types, as TIM-3 expressed on T cells generally mediates inhibitory signals, whereas TIM-3 on myeloid cells has been linked to pro-inflammatory functions in certain contexts (129). These complexities underscore the need for a nuanced understanding of TIM-3 biology and careful ligand-specific therapeutic design in ADs applications.

Therapeutic strategies targeting TIM-3 in AD generally fall into two categories (1): IgG1-type antibodies designed to deplete TIM-3–expressing immune cells, and (2) IgG4 monoclonal antibodies aimed at modulating TIM-3 signaling to restore immune regulation (130). Unlike the inhibitory antibodies employed in oncology, these agents functionally act as agonists of checkpoint pathways, thereby enhancing immune tolerance and suppressing autoimmunity. Such interventions may mitigate immune hyperactivation and alleviate disease manifestations.

Despite these encouraging preclinical findings, clinical evidence remains limited. A major unresolved issue is the ligand promiscuity of TIM-3, which complicates both mechanistic interpretation and therapeutic targeting. Most investigations of TIM-3 in autoimmunity still rely on murine models, which incompletely recapitulate human IC biology. Moving forward, three actions are needed: first, ligand-specific therapeutic design that accounts for TIM-3’s promiscuous interactions (Galectin-9, HMGB1, CEACAM-1, PtdSer); second, systematic evaluation of TIM-3 agonists in human autoimmune tissues using single-cell approaches; and third, well-controlled clinical trials to assess safety and efficacy across different ADs. Until then, the translational promise of TIM-3 remains to be realized.

### 3.4 Lymphocyte activation gene 3

LAG-3 (CD223) was first identified in 1990 as a novel IC receptor. Early investigations faced challenges in defining its functions due to the complexity of ligand interactions and signaling pathways (131, 132). Clarifying its functional ligands has been central to advancing targeted immunotherapy. Initially, MHC-II molecules were considered the canonical ligands; however, subsequent studies revealed that LAG-3 selectively binds to stable peptide–MHC-II complexes rather than universally engaging all MHC-II molecules (133). Later, fibrinogen-like protein 1 (FGL1) was identified as an additional high-affinity ligand, pointing to an immune regulatory pathway distinct from PD-1/PD-L1 and broadening therapeutic opportunities for LAG-3 targeting (134). More recent analyses, however, indicate that stable peptide-MHC-II complexes represent the primary functional ligands mediating LAG-3-dependent immune regulation, while FGL1 appears to have limited inhibitory capacity, thereby resolving a long-standing debate in the field (135). Beyond ligand binding, recent structural and functional studies have suggested an additional layer of regulatory complexity: LAG-3 may modulate T-cell activation through spatial proximity to the TCR complex. This regulation appears to be MHC-II-dependent yet functionally distinct from classical CD4 co-receptor engagement. Building on this concept, emerging strategies have explored bispecific antibodies designed to enforce LAG-3-TCR co-localization, which can attenuate TCR signaling and T-cell activation in preclinical models (136). However, the precise molecular mechanisms and their physiological relevance remain to be fully elucidated.

A critical conceptual advance in understanding LAG-3 function in autoimmunity is its context-dependent regulation of Tregs. Unlike PD-1, which generally enhances Treg suppressive capacity, LAG-3 exhibits dual and seemingly opposing roles depending on the inflammatory milieu. In steady-state conditions, LAG-3 expression on Tregs contributes to their suppressive function, and LAG-3^+^ Tregs produce IL-10 to limit excessive immune responses (137). However, in chronic autoimmune-prone environments such as the non-obese diabetic (NOD) mouse model of T1D, LAG-3 intrinsically limits Treg proliferation and function at inflammatory sites, thereby promoting autoimmunity (138). This paradoxical observation underscores that LAG-3 agonism may not be universally beneficial across all ADs; instead, its therapeutic effect is likely context-dependent and may require careful disease-specific evaluation.

The functional synergy between LAG-3 and PD-1 is well-established. In murine models, combined deficiency of *LAG-3* and *PD-1* results in pronounced immune hyperactivation and spontaneous autoimmunity, demonstrating their non-redundant and cooperative roles in contributing to immune tolerance (139). In the context of T-cell dysfunction, LAG-3 and PD-1 synergistically drive this state while restraining clonal expansion, and deletion of both receptors yields enhanced functional recovery compared to deletion of either alone (140). This synergy has been successfully exploited in oncology, where the combination of nivolumab (anti-PD-1) and relatlimab (anti-LAG-3) is FDA-approved for melanoma (141). In autoimmunity, this same synergy suggests that dual targeting, combining LAG-3 agonism with PD-1 agonism, could produce additive or synergistic immunosuppressive effects, though this approach has not yet entered clinical testing and carries theoretical risks of compounded immune suppression.

LAG-3 plays a central role in immune regulation by modulating T-cell activation, maintaining tolerance, and preventing excessive immune responses. Dysregulation of LAG-3 has been implicated in the development and progression of ADs. In murine models, combined deficiency of LAG-3 and PD-1 results in pronounced immune hyperactivation and spontaneous autoimmunity (139). In RA, LAG-3 expression is reduced, particularly in patients with severe disease, and similar reductions have been observed in psoriasis (142, 143). LAG-3^+^ Tregs have emerged as a novel protective Treg subset that produces IL-10. Alterations in LAG-3^+^ Tregs have been reported in multiple ADs, including RA, psoriasis, and psoriatic arthritis, and may correlate with response to therapy (131). In T1D, *LAG-3* deficiency is associated with accelerated disease onset, with mice exhibiting 100% morbidity, while administration of anti–LAG-3 blocking antibodies further exacerbates disease, emphasizing the checkpoint’s role in restraining autoimmunity (138). Patients with MS and T1D exhibit markedly lower frequencies of LAG-3^+^ CD4^+^ and CD8^+^ T cells in their peripheral blood relative to healthy individuals. The reduced LAG-3 protein levels are attributable to changes in mRNA expression rather than to cleavage of the cell surface receptor (144). Together, these findings highlight the pivotal contribution of LAG-3 to autoimmune pathogenesis and its promise as a therapeutic target.

Interestingly, a meta-analysis of genome-wide association studies for autoimmune thyroid disease (AITD) uncovered a start codon variant in *LAG3* (rs138077246) that correlates with reduced LAG-3 expression and heightened AITD risk, offering direct human genetic evidence that ties LAG-3 deficiency to autoimmunity (145).

Recent translational efforts have sought to develop monoclonal antibodies against LAG-3. One example is GSK2831781, a humanized IgG1 antibody engineered with AFUCO glycosylation to enhance cytotoxicity and efficacy (146). This antibody selectively depletes activated, LAG-3–positive T cells in a dose-dependent manner and has been tested in T-cell–mediated inflammatory disorders such as psoriasis. Clinical evaluation in patients with moderate-to-severe UC was initiated to assess its safety, tolerability, efficacy, and dose response (NCT03893565). However, an interim data analysis led to early termination of the trial, because although GSK2831781 effectively depleted target cells in the bloodstream, it did not alleviate colonic mucosal inflammation, indicating a lack of pharmacological efficacy in UC (147). This failure highlights the challenge of translating LAG-3–targeted strategies from systemic depletion to effective therapy in tissue-specific autoimmune conditions, and underscores the importance of understanding tissue-specific LAG-3 biology.

A more recent and conceptually distinct approach involves the development of bispecific antibodies that enforce spatial proximity between LAG-3 and the TCR complex. Du et al. reported that a bispecific antibody artificially bringing LAG-3 into close proximity with the TCR suppresses both CD4^+^ and CD8^+^ T-cell activation and effectively alleviates autoimmune symptoms in multiple mouse models, including EAEs and collagen-induced arthritis (CIA). Unlike conventional PD-1 agonists, this inhibition is independent of CD4 co-receptors and achieves potent suppression without broad immunosuppression (136). This represents a promising new direction for LAG-3-targeted therapy in ADs, though clinical translation remains pending.

Several critical limitations temper the therapeutic promise of LAG-3 targeting in ADs. One concern is the context-dependent behavior of LAG-3 on regulatory T cells (Tregs): although it can enhance suppression under steady-state conditions, it may constrain Treg proliferation in inflammatory milieus such as T1D. Another obstacle is the translational disconnect observed with GSK2831781, which depleted circulating LAG-3^+^ cells but failed to reduce colonic mucosal inflammation—a reminder that systemic target engagement does not guarantee tissue efficacy. Moreover, most mechanistic insights derive from murine models, raising questions about their relevance to human disease. Furthermore, the promiscuity of LAG-3 ligand interactions (including MHC-II, FGL1, galectin-3, and LSECtin) introduces a risk of unintentionally activating pro-inflammatory signals, underscoring the need for careful ligand-selective therapeutic design (148, 149).

Moving forward, coordinated advances should focus on mapping tissue-specific LAG-3 functions, designing bispecific agonists that enforce LAG-3-TCR co-localization, and identifying soluble LAG-3 as a circulating biomarker (150).

### 3.5 T cell immunoreceptor with Ig and ITIM domains

TIGIT is a co-inhibitory receptor expressed on activated T cells, Tregs, and NK cells, and has also been reported on a subset of B cells. TIGIT binds to CD155 (PVR) and CD112 (PVRL2) on APCs and exerts immunomodulatory effects through multiple mechanisms, including intrinsic inhibitory signaling mediated by its cytoplasmic motifs, competition with the co-stimulatory receptor CD226 for ligand binding, and the induction of tolerogenic phenotypes in DCs (151, 152). Genetic and transcriptional studies further suggest the involvement of the TIGIT/CD226 axis in susceptibility to ADs, including MS and RA (153). Consistent with this, *TIGIT*-deficient mice exhibit enhanced T cell activation and increased susceptibility to autoimmune responses, supporting an important, albeit context-dependent, role in contributing to immune tolerance (152).

A distinguishing feature of TIGIT in the regulation of autoimmune inflammation is its preferential suppression of pro-inflammatory Th1 and Th17 responses. TIGIT^+^ Tregs have been shown to preferentially suppress Th1 and Th17 cells while relatively sparing Th2 responses, thereby contributing to a shift toward Th2-skewed immunity in specific settings (154). In experimental autoimmune uveitis (EAU), a recent study reported that TIGIT stimulation at the onset of clinical symptoms reduced disease severity and limited Th17 cell infiltration into the eye. Moreover, Tregs derived from TIGIT agonist–treated mice were capable of transferring protection to recipient animals, suggesting that TIGIT agonism may promote durable regulatory immune responses (155). Similarly, in a mouse model of collagen-induced arthritis (CIA), TIGIT overexpression was shown to attenuate CD4^+^ T cell effector function and ameliorate disease severity (156).

In the context of human ADs, emerging evidence points to the TIGIT/CD226/CD155 axis as an important regulator of T cell responses. The TIGIT/CD226 axis is differentially expressed in MS, and alterations in this pathway have been associated with disease susceptibility (153). In SLE, TIGIT signaling has been reported to negatively regulate CD4^+^ T-cell responses, although its relationship with disease activity appears to be context-dependent and remains incompletely defined (157). More recently, circulating TIGIT^+^PD-1^+^ T peripheral helper (TPH) and T follicular helper (TFH) cells were found to be increased in SLE patients and positively correlated with autoantibody production and disease severity, whereas TIGIT^+^PD-1^-^ TFH cells were reduced and inversely associated with disease activity. Logistic regression analysis further identified TIGIT^+^PD-1^+^ TPH and TFH subsets as independent risk factors for SLE, suggesting that distinct TIGIT/PD-1 expression patterns on T helper subsets may serve as potential predictors of disease activity (158).

The interaction between TIGIT and DCs adds an additional layer of complexity to its immunoregulatory function. Engagement of CD155 on DCs by TIGIT-expressing cells has been shown to induce an immunoregulatory DC phenotype, characterized by increased IL-10 production, reduced expression of co-stimulatory molecules, and decreased IL-12 secretion (151). While this mechanism is well established in preclinical systems, including *in vitro* and murine models, direct evidence in human AD settings remains limited. For example, in psoriatic disease, increased CD155 expression on myeloid and plasmacytoid DCs has been associated with inflammatory activity, suggesting involvement of the TIGIT/CD155 axis in human immune dysregulation (159), although such observations do not directly establish TIGIT-mediated tolerogenic signaling. Nevertheless, therapeutic exploitation of this pathway has shown promise: a recombinant TIGIT-Fc fusion protein has been reported to induce tolerogenic antigen-presenting cells and Tregs in preclinical models (151, 160). Additionally, a recombinant adenoviral vector (Adv5) designed to generate TIGIT-expressing B cells has demonstrated preclinical efficacy in controlling inflammatory T cell responses in an EAEs model, an effect attributed to engagement of CD155 on DCs and modulation of T cell function (161). Collectively, these findings support the TIGIT–CD155 axis as a potential target for promoting immune tolerance, while underscoring the need for further mechanistic validation and translational studies in human ADs.

TIGIT also exhibits functional interplay with PD-1, although the relative contribution of each pathway appears to be highly context- and disease-dependent. In autoimmune diabetes models, TIGIT is highly expressed on islet-infiltrating T cells, yet TIGIT blockade alone does not markedly exacerbate disease, whereas PD-1/PD-L1 blockade rapidly accelerates disease onset. Notably, combined perturbation of PD-1 signaling and TIGIT inhibition has been reported to further enhance disease progression, suggesting a cooperative or partially redundant relationship between these pathways in certain settings (162). These findings imply that combinatorial targeting strategies involving TIGIT and PD-1 may represent a potential avenue for modulating immune responses in selected contexts.

Preclinical and early translational studies have explored the therapeutic potential of TIGIT agonism. In these studies, a human agonistic anti-TIGIT monoclonal antibody attenuated CD4^+^ T cell activation, notably in TIGIT^+^ TFH and TPH subsets, and augmented the suppressive function of naïve Tregs, suggesting that TIGIT agonism can restore immune balance in autoimmune conditions (163). Beyond antibody-based approaches, the aforementioned adenoviral vector strategy represents a distinct modality for enforcing TIGIT-mediated immunosuppression (161). However, TIGIT agonists have not yet progressed to well-established clinical evaluation in ADs. In contrast, extensive clinical development of TIGIT antagonists in oncology has yielded mixed results, with several late-stage trials reporting limited efficacy (164).

Several critical limitations also temper the therapeutic promise of TIGIT targeting in ADs. A central challenge in targeting TIGIT lies in its context-dependent expression and functional redundancy with co-inhibitory receptors such as PD-1. Although preclinical studies show efficacy in uveitis, arthritis and EAE models, no robust human data exist. The functional interplay with PD-1 adds further complexity, as the relative importance of each pathway may vary across diseases. Furthermore, reports on TIGIT expression in autoimmune patients are inconsistent, showing elevation in some studies and reduction in others, underscoring the need for disease stage-specific analyses that account for differences in immune cell subsets and treatment status.

Progress will depend on understanding tissue-specific TIGIT functions, developing optimized agonists (e.g., multivalent formats), and identifying predictive biomarkers such as TIGIT/CD226 ratios.

### 3.6 Other ICs in ADs

Beyond CTLA-4, PD-1, LAG-3, TIM-3, and TIGIT, several additional immune regulatory pathways have been implicated in ADs, although most remain in preclinical or early translational stages. These molecules can be broadly grouped into co-inhibitory receptors and co-stimulatory pathways, reflecting their distinct immunological roles.

#### 3.6.1 Co-inhibitory receptors

VISTA (V-domain Ig suppressor of T cell activation) is a hematopoietic checkpoint molecule that can function as both a receptor and a ligand and maintains T cell quiescence and peripheral tolerance. Preclinical studies in murine inflammatory disease models suggest that VISTA signaling restrains autoimmune pathology, whereas its deficiency exacerbates disease severity (165). However, its role in human autoimmune tissues remains incompletely defined, and no clinical translation in ADs remains very limited, with no advanced-stage trials reported to date.

BTLA (B and T lymphocyte attenuator) interacts with herpesvirus entry mediator (HVEM) to suppress T and B cell activation and maintain immune homeostasis. *BTLA*-deficient mice develop exacerbated autoimmune phenotypes in models of EAEs and colitis (166). Nevertheless, therapeutic translation remains limited due to partial functional redundancy with other inhibitory receptors (e.g., PD-1, LAG-3), particularly in regulating T-cell activation thresholds, as well as the complexity of HVEM-mediated signaling networks.

#### 3.6.2 Co-stimulatory pathways

CD40/CD40L (CD40 ligand, also known as CD154) axis is a central driver of T cell-dependent B cell activation and autoantibody production. Preclinical studies demonstrate its importance in systemic autoimmunity, and blockade has shown efficacy in lupus and transplantation models. However, early clinical development of anti-CD40L antibodies was limited by thromboembolic complications. Next-generation agents, including Fc-modified anti-CD40L antibodies and CD40-targeting strategies, have re-entered early-phase clinical evaluation, but definitive efficacy and safety remain to be established (167).

OX40/OX40L (tumor necrosis factor receptor superfamily member 4/its ligand) pathway promotes T cell expansion, T follicular helper cell differentiation, and memory formation. Preclinical models support its role in autoimmune inflammation. Studies have shown that synovial macrophage OX40L expression contributes to Tfh cell differentiation in the joint microenvironment, and higher levels of synovial macrophage OX40L expression are associated with enhanced Tfh cell development, thereby contributing to the pathogenesis of RA (168). However, clinical trials in ADs have yielded inconsistent results, with clinical studies in rheumatoid arthritis not consistently demonstrating clear therapeutic benefit. These outcomes may reflect pathway redundancy and disease-specific immune architecture.

ICOS/ICOSL (inducible T cell costimulator/its ligand) signaling is essential for T follicular helper cell differentiation and germinal center responses. Preclinical lupus models suggest that ICOSL blockade reduces autoantibody production. However, dual roles of ICOS in effector and Treg subsets, combined with redundancy with CD28 signaling, have limited clinical translation, and robust efficacy has not yet been confirmed in late-stage clinical trials in ADs (169).

### 3.7 Common limitations and future directions

Across these pathways, several shared limitations exist. First, most evidence is derived from murine models, and human validation using single-cell and spatial transcriptomic approaches remains insufficient. Second, functional redundancy among immune checkpoints suggests that single-target strategies may have limited efficacy. Third, clinical translation has been challenging, with several pathways showing limited or unproven efficacy in early trials. Finally, long-term safety of immune modulation in chronic ADs remains poorly characterized.

Future studies should focus on human tissue validation, development of multi-target or bispecific strategies, identification of predictive biomarkers, and systematic evaluation of long-term safety.

## 4 Combination immunotherapy approaches

**Figure 5:**
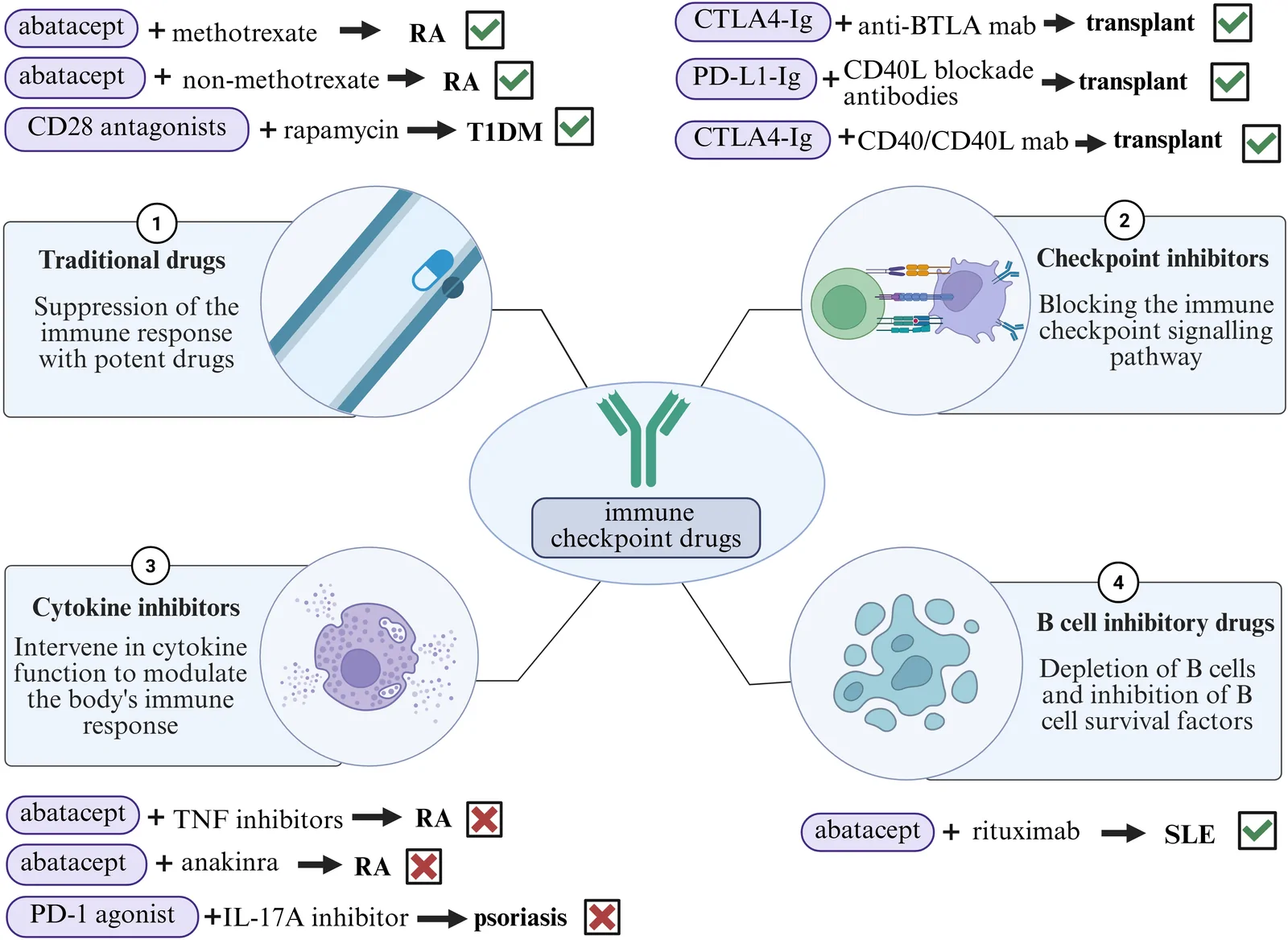
Strategies for the treatment of ADs in combination with ICs drugs. There are four main strategies used in combination with ICs drugs for the treatment of ADs, suppressing the immune response through different mechanisms.

In certain clinical scenarios, some patients with ADs fail to achieve adequate responses with monotherapy, prompting consideration of combination treatment strategies (**Figure 5**). Combination therapy can enhance efficacy, reduce the development of drug resistance, enable personalized treatment approaches, and more comprehensively manage both disease symptoms and progression. However, this approach also carries an increased risk of adverse effects, making safety considerations paramount. Because most AD therapies aim to suppress overactive immune responses, concurrent intervention in multiple immune pathways may further elevate susceptibility to infections.

A critical distinction must be made between pursuing synergistic clinical efficacy and inadvertently incurring synergistic immunotoxicity. The benefit-risk ratio of combination therapy depends on whether additive or synergistic effects can be achieved without compounding toxicities. If efficacy is primarily driven by blocking or agonizing a single pathway, dual targeting may not provide additional benefit while increasing adverse events. Furthermore, much of the preclinical rationale for combination immunotherapy derives from murine transplant models of acute alloreactivity. These findings cannot be directly extrapolated to chronic, self-antigen-driven autoimmunity, as the two settings differ fundamentally in T-cell repertoire diversity, antigen persistence, duration of inflammation, and regulatory network engagement. Recognizing this translational gap is essential when interpreting promising combination data from transplantation studies. For example, in methotrexate-resistant RA, the combination of etanercept and anakinra did not improve outcomes compared to etanercept alone, but led to higher infection rates (170). Similarly, a phase 2 study of the bispecific antibody ABT-122, which simultaneously inhibits tumor necrosis factor (TNF) and IL-17A, found comparable efficacy and safety to TNF inhibition alone, with no significant superiority over adalimumab (171). These findings underscore the importance of rationally designing combination strategies to maximize therapeutic gain while avoiding unnecessary adverse reactions.

### 4.1 ICs-targeted agents combined with traditional drugs

Conventional treatments for ADs typically involve potent immunosuppressive agents aimed at dampening overactive immune responses. While effective, these therapies often carry significant risks, including increased susceptibility to infections and, in some cases, malignancies. In contrast, ICs–targeted therapies offer a more precise approach by selectively modulating specific molecular targets within the immune system. Combining ICs-targeted agents with traditional therapies has been explored as a strategy to enhance clinical benefits while potentially mitigating adverse effects.

In 2010, the European Medicines Agency approved the combination of abatacept with methotrexate for patients who failed to respond adequately to at least one disease-modifying anti-rheumatic drug and one TNF-α inhibitor (172). Clinical studies have demonstrated that this combination improves outcomes in RA. For example, abatacept plus methotrexate has been shown to reduce disease activity, improve clinical manifestations associated with rheumatoid factor positivity, and slow the progression of early joint lesions, thereby preserving joint function (173). Comparable benefits have been observed when abatacept is combined with non-methotrexate disease-modifying anti-rheumatic drug, indicating flexible potential in RA management (174).

Preclinical studies further illustrate the rationale for combination strategies. In a murine model of autoimmune diabetes, co-administration of CD28 antagonists with rapamycin delayed disease progression through complementary mechanisms, demonstrating that targeting distinct immunoregulatory pathways can yield cooperative effects (175).

However, not all combination approaches guarantee improved outcomes. In lupus nephritis, adding abatacept to a regimen of low-dose cyclophosphamide and azathioprine did not produce significant clinical improvement, although the therapy was well tolerated (176). This observation may reflect the limited efficacy of abatacept at the doses tested, which were based on RA treatment regimens, suggesting that higher or optimized dosing may be required in lupus nephritis. Systematic investigations into dosing, timing, and combination protocols are necessary to fully evaluate abatacept’s therapeutic potential in this context. Such studies could provide a more comprehensive understanding of the balance between efficacy and safety, guiding rational clinical application.

A key lesson from failed combinations is that adding an IC-targeted agent to a traditional immunosuppressant is not automatically beneficial. The lack of efficacy in lupus nephritis and the increased infection risk observed with certain combinations (discussed in Section 4.3) underscore the need for rigorous preclinical modeling that recapitulates the chronic, tissue-specific nature of human ADs, rather than relying solely on acute inflammatory or transplant models.

### 4.2 ICs-targeted agents combined with ICs-targeted agents

Although monotherapy with IC agonists has shown clinical benefit in some patients, dual targeting of multiple pathways may amplify therapeutic efficacy through synergistic effects, providing a rationale for refined combination strategies. However, much of the preclinical evidence for combination IC strategies derives from murine models of transplantation, where the goal is to prevent allograft rejection—an acute response against strong alloantigen. In contrast, chronic ADs involve persistent, low-affinity self-antigen stimulation, a distinct T-cell repertoire, and different regulatory dynamics. Therefore, positive results from transplant models cannot be directly extrapolated to ADs without careful validation. Recognizing this translational gap is essential when interpreting the following studies.

In murine graft models, dual blockade strategies have shown remarkable promise. For example, administration of CTLA4-Ig or an anti-BTLA monoclonal antibody alone modestly extended graft survival, whereas their combination achieved indefinite graft survival, with some grafts persisting for over 100 days (177). Similarly, in islet transplantation models, PD-L1-Ig combined with CD40L blockade enabled long-term pancreatic islet survival, whereas either agent alone failed to prevent rejection (178). A particularly well studied approach is the combination of CTLA4-Ig, which blocks the CD28 pathway, with antibodies targeting CD40/CD40L. In experimental systems, dual blockade of these pathways effectively suppressed T-cell clonal expansion, prolonged skin and cardiac allograft survival, and reduced chronic vascular rejection (179, 180). Additional work demonstrated that CTLA4-Ig in combination with anti-CD40 monoclonal antibodies such as 7E1-G2b or 3A8 not only improved graft survival but also prevented the development of donor-specific antibodies following islet transplantation, supporting durable graft tolerance (181, 182). Notably, in rhesus monkey kidney allografts, co-administration of CTLA4-Ig and the CD40L-specific antibody 5C8 exhibited up to 100-fold greater efficacy compared with monotherapy, further highlighting the therapeutic potential of this strategy (183).

Despite these compelling results in transplantation, direct evidence for synergistic efficacy of dual IC targeting in spontaneous ADs models remains limited. Few studies have systematically tested combinations of IC agonists (e.g., CTLA-4-Ig plus PD-1 agonist) in chronic autoimmune settings such as lupus-prone mice or NOD mice. Moreover, the risk of compounded immunosuppression, including increased susceptibility to infections and potential impairment of tumor surveillance, has not been adequately evaluated in long term studies. Thus, while dual IC targeting represents an intellectually attractive strategy, its clinical translation for ADs will require dedicated autoimmune models, careful dose optimization, and rigorous safety assessments that go beyond transplantation data.

Overall, combining IC-targeted agents represents an emerging therapeutic paradigm aimed at fine-tuning immune responses in ADs through simultaneous inhibition or activation of multiple checkpoint pathways. The principal advantages of this approach include synergistic efficacy, higher clinical response rates, and the potential to overcome drug resistance. Nevertheless, the increased risk of immune-related toxicity and the substantial translational gap between transplant models and chronic autoimmunity remain significant concerns. Future research should prioritize head-to-head comparisons of dual versus monotherapy in spontaneous autoimmune models, along with pharmacokinetic and pharmacodynamic studies to define optimal dosing regimens that maximize efficacy while minimizing additive toxicities.

### 4.3 ICs-targeted agents combined with cytokine inhibitors

Cytokines are protein mediators that orchestrate intercellular communication and play pivotal roles in immune regulation, inflammation, and tissue homeostasis. Dysregulated cytokine signaling is a hallmark of many ADs. For instance, in RA and IBD, excessive production of pro-inflammatory cytokines such as TNF-α drives chronic inflammation and tissue damage (184, 185). Accordingly, cytokine-targeted therapies, particularly TNF-α inhibitors, have become a cornerstone in the management of these conditions.

Given the complementary mechanisms of action, combining IC modulators with cytokine inhibitors has been explored as a strategy to enhance therapeutic efficacy. However, safety concerns have emerged. Clinical studies indicate that the co-administration of abatacept with TNF inhibitors or with the IL-1 receptor antagonist anakinra is associated with an elevated risk of both overall and severe infections compared with abatacept plus placebo (186, 187). These findings suggest that although abatacept provides effective costimulatory blockade, concurrent suppression of cytokine pathways may excessively compromise host defense. For this reason, current clinical guidelines advise against such combinations, and careful monitoring for infectious complications is essential during transitions from TNF inhibitor therapy to abatacept.

More recent attempts to combine IC-targeted agents with cytokine inhibitors have encountered further challenges. A phase 1 trial investigated a PD-1 agonist (peresolimab, LY3462817) in combination with an IL-17A inhibitor (LY3509754) for psoriasis (NCT04152382). Although the approach was designed to synergistically modulate T-cell activity and dampen IL-17-driven inflammation, hepatotoxicity was observed during the study, raising concerns regarding the tolerability of this combination (188). This example illustrates that even when individual agents are well tolerated, their combination can produce unpredicted organ-specific toxicities. Beyond infection risk and hepatotoxicity, additional safety concerns exist; these broader immunotoxicity issues are discussed in the dedicated irAE section.

Collectively, while cytokine inhibitors and IC-targeted agents each offer validated benefits in ADs, their concurrent use introduces safety challenges, as detailed in Section 5. A critical lesson is that biological synergy at the pathway level does not guarantee clinical synergy; instead, it may unmask additive toxicities. Future research should focus on: (1) delineating the mechanistic basis of deleterious interactions using chronic autoimmune models; (2) identifying biomarkers that predict safe and effective combinations, such as baseline inflammatory profiles or genetic variants affecting drug metabolism; and (3) designing sequential rather than simultaneous dosing regimens to separate efficacy from toxicity. Until such data are available, the combination of IC-targeted agents with cytokine inhibitors should be approached with caution and reserved for well-monitored clinical trials.

### 4.4 ICs-targeted agents combined with B cell inhibitory drugs

B cells play a multifaceted and essential role in the pathogenesis of ADs (189, 190). They contribute to disease progression through the production of pathogenic autoantibodies that erroneously recognize self-antigens, causing direct tissue injury and inflammation. In addition, autoantibodies can form immune complexes with self-antigens, which subsequently deposit in tissues and activate inflammatory cascades, resulting in further organ damage.

**Figure 6:**
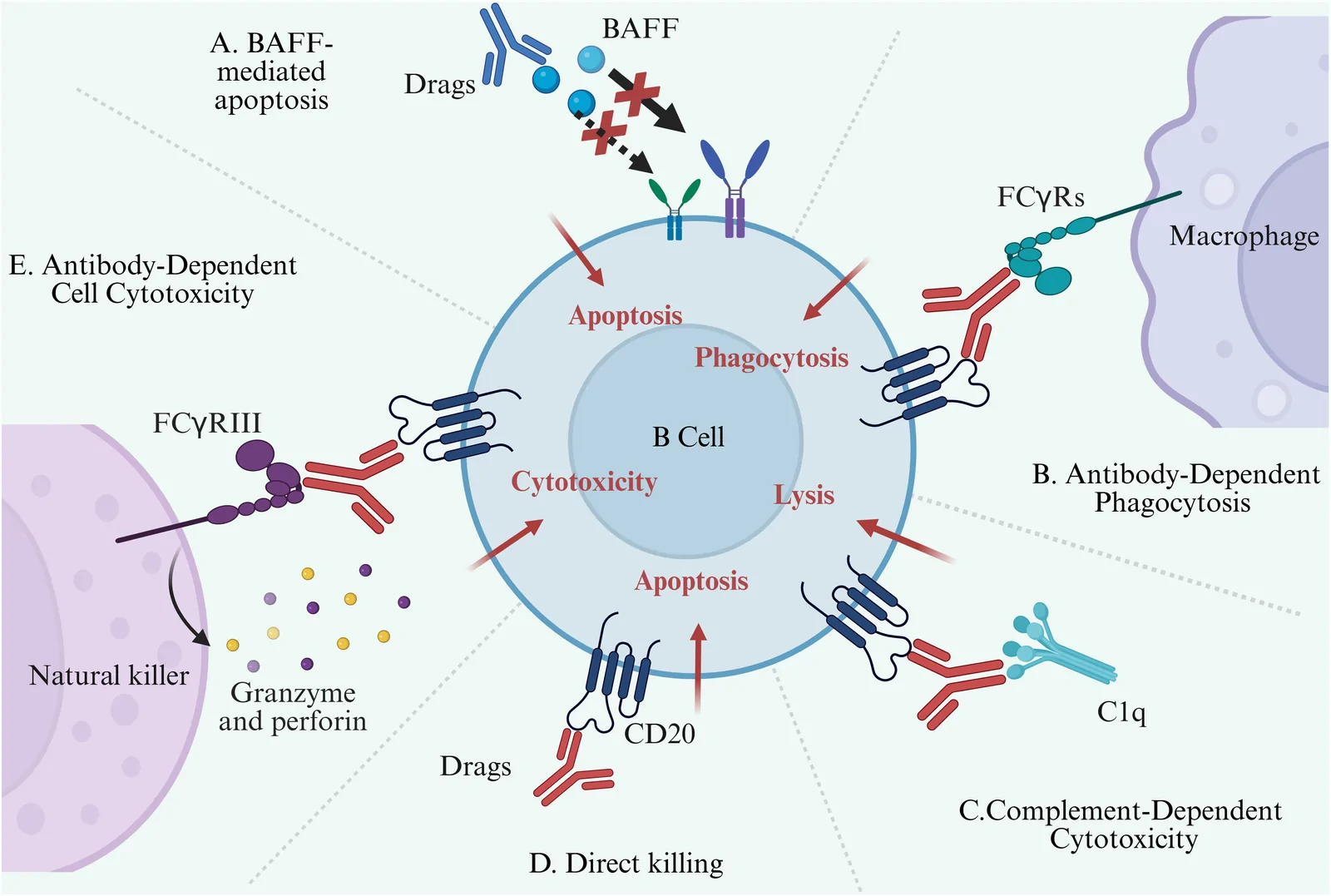
B-cell clearance strategies. B-cell clearance strategies can induce B-cell apoptosis by recognizing and acting on specific cytokines that maintain B-cell survival, such as B-cell activation factor (BAFF). Alternatively, the use of antibody drugs targeted to bind B-cell specific antigens kills B cells through antibody-dependent cell cytotoxicity (FCγRIII binds to drug-CD20, initiating a signaling pathway that leads to the release of granzymes and perforin, thereby killing the B cells) or complement-dependent cytotoxicity (C1q binds to the Fc region of drug-CD20, initiating the classical pathway that ultimately leads to cell lysis) that mediates B cell lysis. B cells are cleared through antibody-dependent phagocytosis (the macrophage FCγ receptor recognizes drug-CD20, initiating a signaling pathway that leads to phagocytosis of the B cell) and direct killing (the drug binds to CD20-expressing B cells, initiating a downstream signaling pathway that leads to apoptosis).

Therapeutic strategies directed against B cells have become a cornerstone of AD management. The most established approaches involve B-cell depletion through monoclonal antibodies such as belimumab and rituximab (**Figure 6**). Belimumab inhibits the binding of the B-cell activating factor, a cytokine critical for B-cell survival and differentiation, thereby reducing autoreactive B-cell persistence (191). Rituximab, on the other hand, selectively targets the CD20 antigen expressed on B cells, leading to their depletion and consequently dampening autoantibody production and inflammation (192).

Beyond conventional monoclonal antibody therapy, engineered T-cell–based approaches have been developed to achieve targeted B-cell elimination. Chimeric antigen receptor (CAR) T-cell immunotherapy, originally pioneered for hematological malignancies, has recently been adapted for the treatment of ADs. CAR T cells engineered to recognize the B-cell marker CD19 have demonstrated promising efficacy in several conditions (193). In 2021, a landmark case demonstrated the therapeutic potential of this approach when anti-CD19 CAR T-cell therapy was administered to a 20-year-old patient with refractory SLE, leading to rapid disease remission without major adverse effects (194). Building on this, follow-up studies reported that five patients with refractory SLE achieved sustained, drug-free remission for up to 17 months after CD19-directed CAR T-cell therapy, without clinical relapses (195). In 2023, the same group successfully applied this strategy in a patient with severe anti-synthetase syndrome, who remained in remission six months after treatment, without requiring immunosuppressants (196). More recently, clinical application has been extended to B-cell–driven MS. In this setting, treatment with KYV-101, a fully humanized CD19 CAR construct with rationally designed co-stimulatory domains, effectively suppressed relapses and eliminated residual B cells within the central nervous system that are implicated in disease persistence (197). Additionally, bispecific T cell engagers (BiTEs), which redirect T cells to lyse target cells, have emerged as a promising strategy for refractory ADs. For example, the CD19×CD3 BiTE blinatumomab induced profound B cell depletion and clinical remission in patients with multidrug-resistant RA, including those who had failed rituximab therapy (198). Similarly, the BCMA×CD3 BiTE teclistamab effectively targeted plasma cells—which often express BCMA but not CD19—in patients with systemic sclerosis, Sjögren’s syndrome, myositis, and RA, leading to reduced autoantibody levels and improved disease activity (199). These approaches leverage cytotoxic T cells to achieve deep B cell and plasma cell depletion, offering a complementary mechanism to CAR T-cell therapy. Collectively, these examples highlight the transformative potential of CAR T-cell therapy and T-cell engagers in ADs. Nevertheless, its clinical use remains in early stages; long-term data on safety, durability, and relapse risk are limited, and ongoing research is focused on addressing these challenges (200).

When considering the combination of IC-targeted agents with B-cell inhibitory drugs, several mechanistic rationales and safety considerations emerge. Theoretically, simultaneous targeting of T cell co-stimulation (e.g., with abatacept) and B cell depletion (e.g., with rituximab) could produce additive or synergistic effects by interrupting two interdependent arms of the autoimmune response. In contrast to the combination of IC-targeted agents with cytokine inhibitors (Section 4.3), which has shown increased infection risk, the combination of abatacept and rituximab has been reported to be relatively well tolerated in small case series. For example, dual administration of rituximab and abatacept improved clinical symptoms in patients with SLE and mixed connective tissue disease, suggesting potential additive benefits (201). A clinical trial was subsequently initiated to evaluate this combination in the context of T1D (NCT03929601), aiming to mitigate autoimmune pathology through complementary mechanisms (202). However, the trial was suspended before completion, and no definitive conclusions can be drawn.

By contrast, dual treatment with belimumab and rituximab in lupus nephritis was reported to be well tolerated and did not exacerbate adverse events compared with either agent alone, although clear superiority over monotherapy has not been established (203). In clinical practice, rituximab is often used to reduce the reliance on long-term corticosteroids, such as prednisone, thereby limiting steroid-related toxicities. Thus, integrating IC modulators with B cell inhibitory therapies may provide an additional therapeutic avenue, particularly for refractory lupus nephritis, but evidence of added benefit over monotherapy remains inconclusive.

Several critical limitations must be acknowledged. First, the combination of IC-targeted agents with B-cell depletion has not been rigorously tested in large randomized controlled trials, and most evidence comes from small case series or terminated studies. Second, the theoretical risk of compounded immunosuppression, including prolonged B cell aplasia, hypogammaglobulinemia, and increased susceptibility to infections, has not been systematically evaluated. Third, the optimal sequencing and dosing of these combinations are unknown; for example, it is unclear whether B-cell depletion should precede or follow IC modulation. Fourth, the long-term risk of malignancies, particularly lymphoproliferative disorders, remains a concern when combining agents that affect both T-cell and B-cell compartments.

Looking forward, advancing combination strategies with IC-targeted agents and B cell inhibitory drugs will require: (1) well-powered randomized controlled trials with active comparator arms to establish superiority over monotherapy; (2) mechanistic studies to identify biomarkers (e.g., baseline autoantibody profiles, B cell subset composition, or T cell activation status) that predict response; (3) pharmacokinetic and pharmacodynamic investigations to define optimal dosing and sequencing; and (4) long-term safety registries to monitor for infections, hypogammaglobulinemia, and secondary malignancies. Until such data are available, this combination should be reserved for patients with refractory disease in the context of clinical trials or under close supervision.

## 5 Immune-related adverse events: mechanisms, clinical risk–benefit, and implications for autoimmune therapy

A critical consideration for any IC-based therapy is the potential for irAEs. Although irAEs are most extensively documented in oncology following IC blockade, they provide essential insights into the safety landscape of IC agonism in ADs. irAEs are defined as organ-specific or systemic toxicities arising from immune overactivation and the disruption of self-tolerance, fundamentally representing autoimmune-like pathological damage. Clinically, irAEs can be broadly categorized into endocrine (e.g., thyroiditis, autoimmune diabetes), gastrointestinal (e.g., colitis), dermatologic, rheumatologic, and less commonly neurological or cardiovascular manifestations, reflecting widespread immune dysregulation across tissues (204). Notably, irAEs induced by anti-PD-1/CTLA-4 therapy frequently mimic classic spontaneous autoimmune conditions, including thyroiditis, colitis, arthritis, and lupus-like syndromes (205). This phenotypical resemblance suggests shared mechanisms of immune dysregulation, primarily driven by ICI-activated CD8^+^ cytotoxic T cells and, in some cases, by B cells producing pathogenic autoantibodies (206). T-cell subset dysfunction (e.g., autoreactive T-cell activation, Treg imbalance, helper T cell and memory T cell dysfunction) and cytokine network dysregulation (e.g., TNF-α, IL-6, IL-17 pathways) form the core molecular basis of irAEs, with affected tissues exhibiting dysregulated T-cell activity and autoantibody production (207).

The mechanistic overlap between irAEs and spontaneous autoimmunity has been substantiated by studies using single-cell omics and genetic analyses, which have identified both common and distinct T-cell and autoantibody signatures (208). For instance, ICI-induced inflammatory arthritis is immunologically distinct and likely driven predominantly by T cells rather than autoantibodies (209), whereas ICI-induced thyroiditis shares key features with Hashimoto’s thyroiditis (210). The parallels between irAEs and endogenous ADs offer a unique opportunity: by studying irAEs triggered by IC blockade, one can glean mechanistic insights into the failures of immune tolerance that are applicable to AD pathogenesis. Conversely, as IC agonism advances toward clinical application in ADs, these oncology-derived safety data inform a shift in the dominant risk profile—from immune overactivation–driven irAEs to immune suppression–associated complications.

From a risk-benefit perspective, the therapeutic index of IC-based therapies in ADs depends on two opposing immunological axes. On one hand, immune overactivation, characteristic of IC blockade in oncology, can lead to irAEs that phenocopy ADs. On the other hand, immune suppression, more relevant to IC agonism in ADs, increases the risk of infections, may impair tumor surveillance, and can contribute to complications such as hypogammaglobulinemia (particularly in the context of B-cell-depleting strategies) (211). This distinction underscores that the safety profile of IC agonists is fundamentally different from that of IC inhibitors. Accordingly, rigorous long-term safety monitoring in AD trials should prioritize surveillance for opportunistic infections, viral reactivation, and secondary malignancies, in addition to classical autoimmune-like toxicities. Predictive biomarkers, such as pre-existing autoantibodies, baseline cytokine profiles (e.g., IFN-γ, TNF-α, IL-6), T cell subset composition, and genetic risk alleles (e.g., HLA variants or interferon signatures), are emerging as tools to stratify patients and guide risk-adapted monitoring (212). As IC agonism progresses toward clinical use, prospective safety registries and adaptive trial designs will be essential to capture long-term toxicities, define the true risk-benefit balance, and develop mitigation strategies such as dose optimization, sequential therapy, and pre-emptive antimicrobial or immunoglobulin support. Collectively, irAEs should not be viewed solely as adverse outcomes but rather as a translational bridge linking cancer immunotherapy and autoimmunity, providing critical mechanistic insights for both disease domains.

## 6 Discussion and outlook

ADs encompass a broad spectrum of chronic disorders characterized by dysregulated immune responses that result in progressive tissue damage and impaired organ function. The rising prevalence of ADs, particularly among women, highlights a growing public health concern (213). Clinically, ADs pose significant treatment challenges, frequently necessitating long-term or lifelong medication. A central therapeutic goal is the restoration of durable immune tolerance without compromising host defense. Over the past twenty years, a fundamental transition has occurred in the treatment landscape, moving from traditional immunosuppression to precision immunomodulation, with IC-based interventions representing a mechanistically rational strategy.

Several landmark achievements have reshaped the therapeutic landscape. The clinical validation of CTLA-4-Ig (abatacept) in RA, JIA, and psoriatic arthritis, along with promising results in T1D, has established that agonizing a co-inhibitory IC can safely ameliorate autoimmune pathology in humans. More recently, the phase 2 success of peresolimab, a PD-1 agonist, in RA provides the first proof-of-concept that direct activation of PD-1 signaling is feasible and clinically beneficial, opening a new class of therapeutics (103). Beyond protein-based therapies, the repurposing of cell-based approaches from oncology, particularly CD19-directed CAR-T cells and BiTEs, has produced unprecedented, drug-free remissions in refractory SLE, anti-synthetase syndrome, and RA (195, 196, 198). These successes challenge the traditional goal of symptom control and suggest that deep immune resetting may be achievable in selected patients. Meanwhile, the expansion of the IC target landscape beyond CTLA-4 and PD-1, including LAG-3, TIM-3, and TIGIT, has generated a rich pipeline of preclinical and early clinical candidates, each with distinct mechanisms and potential disease-specific niches.

### 6.1 Key challenges and unresolved issues

Despite these advances, several critical hurdles impede broader clinical translation. A major pharmacological challenge lies in the inherent difficulty of IC agonism. Unlike receptor blockade, effective agonism often requires multivalent ligand crosslinking, ITIM/ITSM phosphorylation, and recruitment of phosphatases such as SHP-1 and SHP-2. Most current agonists are bivalent antibodies that may not achieve sufficient receptor clustering, contributing to high attrition rates in clinical development (42). Safety considerations also remain a fundamental constraint. While IC blockade is associated with irAEs, as discussed in the dedicated safety section, IC agonism shifts the dominant risk profile toward immunosuppression-related complications, including infection and impaired tumor surveillance. A major translational gap remains between murine models (particularly acute transplant models) and human ADs, which differ in T-cell repertoire, antigen persistence, and regulatory networks. Another persistent obstacle is the lack of validated predictive biomarkers, which continues to hinder patient stratification and response prediction. Finally, it remains uncertain whether IC dysregulation represents a primary driver of disease or a secondary modulatory layer in most ADs; while rare monogenic IC deficiencies (e.g., CTLA-4 haploinsufficiency) demonstrate causality, they are not representative of the majority of patients (27).

### 6.2 Future perspectives

Looking forward, several priorities will shape the next generation of IC-targeted therapies for ADs. Multi-omics and systems immunology approaches are expected to enable molecular subtyping of ADs, moving beyond clinical phenotypes toward biology-driven stratification, thereby allowing more precise matching of IC-targeted interventions to disease context. On the engineering front, next-generation IC agonists, including multivalent antibodies, bispecific molecules that enforce receptor clustering, and nanoparticle-based platforms, are needed to overcome the pharmacological limitations of current bivalent formats.

Rationally designed combination therapies should be tested in spontaneous autoimmune models before advancing to clinical trials. Priority combinations include PD-1 plus LAG-3 agonism (given their functional synergy) and IC agonists with B-cell-depleting agents (to interrupt interdependent T- and B-cell loops). Equally important, robust clinical trial designs and long-term monitoring frameworks will be essential to evaluate safety and optimize therapeutic windows in IC-based interventions. Beyond these near-term goals, a deeper understanding of immune memory, particularly the role of pathogenic memory stem cells, may eventually allow true cure by eradicating the reservoir of autoreactive lymphocytes. While this goal remains distant, recent CAR-T successes suggest that durable remission without chronic immunosuppression is no longer theoretical.

## 7 Conclusion

In conclusion, IC-based therapies represent a paradigm shift toward mechanism-driven restoration of immune tolerance in ADs. However, key challenges, including pharmacological constraints, context-dependent immune regulation, and safety considerations, must be addressed to enable broader clinical translation. Future progress will depend on advances in precision immunology, next-generation therapeutic design, and biomarker-guided clinical strategies.

## Glossary

ADs: Autoimmune diseases
ICs: Immune checkpoints
NK cells: Natural killer cells
DCs: Dendritic cells
PD-L1: Programmed death ligand 1
TCR: T cell antigen receptor
MHC: Major histocompatibility complex
APC: Antigen-presenting cell
CTLA-4: Cytotoxic T-lymphocyte antigen 4
PD-1: Programmed cell death protein 1
LAG-3: Lymphocyte activation gene 3
AITD: Autoimmune thyroid disease
UC: Ulcerative colitis
TIM-3: T-cell immunoglobulin and mucin domain-3
MS: Multiple sclerosis
IBD: Inflammatory bowel disease
SLE: Systemic lupus erythematosus
RA: Rheumatoid arthritis
SjS: Sjogren’s syndrome
UC: Ulcerative colitis
CD: Crohn’s disease
T1D: Type 1 diabetes
JIA: Juvenile idiopathic arthritis
CNS: Central nervous system
TNF: Tumor necrosis factor
BiTEs: Bispecific T cell engagers
DAMP: Damage-associated molecular pattern
FGL1: Fibrinogen-like protein 1
irAEs: Immune-related adverse events
CAR T-cell: Chimeric antigen receptor T-cell
IRs: Inhibitory receptors
Tregs: Regulatory T cells
Th1: T helper type 1
Th17: T helper type 17
Th2: T helper type 2
Tfh: T follicular helper cells
Tph: T peripheral helper cells
EAE: Experimental autoimmune encephalomyelitis
CIA: Collagen-induced arthritis
NOD: Non-obese diabetic
ITIM: Immunoreceptor tyrosine-based inhibition motif
ITSM: Immunoreceptor tyrosine-based switch motif
HVEM: Herpesvirus entry mediator
VISTA: V-domain Ig suppressor of T cell activation
BTLA: B and T lymphocyte attenuator
ICOS: Inducible T cell costimulator
OX40: Tumor necrosis factor receptor superfamily member 4
